# Dominant Sequences of Human Major Histocompatibility Complex Conserved Extended Haplotypes from *HLA-DQA2* to *DAXX*

**DOI:** 10.1371/journal.pgen.1004637

**Published:** 2014-10-09

**Authors:** Charles E. Larsen, Dennis R. Alford, Michael R. Trautwein, Yanoh K. Jalloh, Jennifer L. Tarnacki, Sushruta K. Kunnenkeri, Dolores A. Fici, Edmond J. Yunis, Zuheir L. Awdeh, Chester A. Alper

**Affiliations:** 1 Program in Cellular and Molecular Medicine, Boston Children's Hospital, Boston, Massachusetts, United States of America; 2 Department of Medicine, Harvard Medical School, Boston, Massachusetts, United States of America; 3 Pulsar Clinical Technologies, Cambridge, Massachusetts, United States of America; 4 Department of Cancer Immunology and AIDS, Dana-Farber Cancer Institute, Boston, Massachusetts, United States of America; 5 Department of Pathology, Harvard Medical School, Boston, Massachusetts, United States of America; 6 Department of Pediatrics, Harvard Medical School, Boston, Massachusetts, United States of America; Fred Hutchinson Cancer Research Center, United States of America

## Abstract

We resequenced and phased 27 kb of DNA within 580 kb of the MHC class II region in 158 population chromosomes, most of which were conserved extended haplotypes (CEHs) of European descent or contained their centromeric fragments. We determined the single nucleotide polymorphism and deletion-insertion polymorphism alleles of the dominant sequences from *HLA-DQA2* to *DAXX* for these CEHs. Nine of 13 CEHs remained sufficiently intact to possess a dominant sequence extending at least to *DAXX*, 230 kb centromeric to *HLA-DPB1*. We identified the regions centromeric to *HLA-DQB1* within which single instances of eight “common” European MHC haplotypes previously sequenced by the MHC Haplotype Project (MHP) were representative of those dominant CEH sequences. Only two MHP haplotypes had a dominant CEH sequence throughout the centromeric and extended class II region and one MHP haplotype did not represent a known European CEH anywhere in the region. We identified the centromeric recombination transition points of other MHP sequences from CEH representation to non-representation. Several CEH pairs or groups shared sequence identity in small blocks but had significantly different (although still conserved for each separate CEH) sequences in surrounding regions. These patterns partly explain strong calculated linkage disequilibrium over only short (tens to hundreds of kilobases) distances in the context of a finite number of observed megabase-length CEHs comprising half a population's haplotypes. Our results provide a clearer picture of European CEH class II allelic structure and population haplotype architecture, improved regional CEH markers, and raise questions concerning regional recombination hotspots.

## Author Summary

The human major histocompatibility complex (MHC) is a gene-dense region highly enriched in immune response genes. MHC genetic variation is among the highest in the human genome and is associated with both tissue transplant compatibility and many genetic disorders. Long-range (1–3 Mb) MHC haplotypes of essentially identical DNA sequence at relatively high (≥0.5%) population frequency (“genetic fixity”), called conserved extended haplotypes (CEHs), comprise roughly half of all European population haplotypes. We sequenced an aggregate of 27 kb over 580 kb in the MHC class II region from *HLA-DQA2* to *DAXX* in 158 European haplotypes to quantify the breakdown of this genetic fixity in the centromeric portion of the MHC and to determine the representative nature within that region of eight previously fully or nearly fully sequenced “common” European haplotypes. We identified the dominant sequences of 13 European CEHs and determined where the “common” sequences did (or did not) represent related CEHs. We found patterns of shared sequence identity among different CEHs surrounded by fixed (for each CEH) but differing sequence. Our direct observational results for population haplotypes explain the mutual occurrence of CEHs and short (5–200 kb) blocks of fixed sequence detected by the statistical measure of linkage disequilibrium.

## Introduction

The human major histocompatibility complex (MHC) is a highly polymorphic genomic region of over 3 Mb on chromosome 6p21. MHC polymorphisms include critical determinants for tissue transplantation success and show strong correlation both with many genetic diseases and with ethnic origin. Haplotype analysis of DNA sequence containing specific allele combinations of two or more nearby genetic loci was first established in the MHC. Many individuals within a human population share a small number of specific MHC haplotypes. These 1 to 3 Mb stretches of nearly identical MHC DNA sequence with high population frequency are called conserved extended haplotypes (CEHs) [1]–[4] or ancestral haplotypes [5], [6]. Virtually all MHC allele-disease associations involve marker alleles of CEHs [2]–[6].

Early work defined CEHs by their alleles at HLA-B, HLA-DR loci and at intermediate MHC genes (i.e., ‘complotypes’ [7]). Later CEH reports extended the core region from *HLA-C* to *HLA-DQB1*. With technological refinements, it became clear individual CEHs carry only one allele (or, rarely, a limited number of variants) at any given locus in this region [2]–[6]; [8]–[16] without apparent recombination. Intervening DNA sequence is therefore essentially conserved (i.e., identical) among the population haplotypes comprising each CEH, and CEHs are essentially identical by descent common population haplotypes. CEH sequence conservation has been verified whenever investigated, whether determined by microsatellite, restriction fragment length polymorphism, dense single nucleotide polymorphism (SNP) or partial resequencing analyses of multiple haplotypes from unrelated individuals [9]–[13], [15]. We have previously referred to the existence of such “fixed” CEH alleles [1]–[4], [8], [14] and the intervening sequence conservation of CEHs as “genetic fixity” [3], [4], [8], [14]–[16].

We define genetic, haplotype or sequence “fixity” to be sequence identity and conservation of a large stretch of genomic sequence, shared by a relatively large number of apparently unrelated individuals, without apparent recombination from an ancestral sequence. By “identity” we mean “essential identity,” thus allowing for minor private mutation or microvariation within individual haplotype sequences comprising a particular CEH (or CEH fragment or block). Several studies have described the extension of genetic fixity in some CEHs telomerically in the class I region to *HLA-A* and centromerically in the class II region to *HLA-DPB1* [1]–[6], [8], [14]–[16]. However, little is known about CEH alleles and conserved dominant sequences between *HLA-DQB1* and *HLA-DPB1* or those centromeric to *HLA-DPB1*. We sought both to confirm the existence of sequence conservation in multiple examples of a wide variety of CEHs and to identify the dominant sequences centromeric to *HLA-DQB1* for the core MHC CEHs most similar to the previously sequenced “common” European haplotypes [17]–[20].

Using consanguineous cell lines, the Wellcome Trust Sanger Institute (http://www.sanger.ac.uk) undertook the MHC Haplotype Project (MHP) [17]–[20] (http://www.ucl.ac.uk/cancer/medical-genomics/mhc) in which eight “common” European MHC haplotypes were fully or nearly fully sequenced over a genomic distance of up to 4.75 Mb. However, no systematic analysis has determined whether these sequences accurately represent the previously established CEHs [1]–[6]. As we argued earlier [4], the extent to which the MHP sequences could be exploited for deciphering genotype-phenotype relationships would require resequencing multiple independent population haplotypes to determine consensus sequence, microvariation, and population representation for each CEH.

Here, we sought to answer two main questions: 1. Are the centromeric portions of the MHP sequences representative of European CEHs? 2. What is the extent of and sequence of a retained dominant sequence centromeric to *HLA-DQB1* for each CEH “represented” by a MHP sequence? We compare the classical HLA markers of the eight MHP sequences with the analogous markers of previously reported European CEHs [3], [4], [6], [21]. Then, we describe partial resequencing of the region centromeric to *HLA-DQB1* of multiple population haplotypes for each CEH these eight sequences putatively represent. The MHC class II region centromeric to *HLA-DQB1* is important both because of its strong association with many complex diseases and its known or suspected recombination hotspots [22], [23]. We document the extent of CEH dominant sequence conservation from *HLA-DQA2* to *DAXX*, and we identify the SNP and deletion-insertion polymorphism (DIP) alleles of the dominant sequence for each CEH. Finally, we identify where MHP sequences accurately represent those dominant sequences, and we discuss several structural and conceptual issues related to local recombination hotspots and linkage disequilibrium (LD).

## Results

### MHP haplotypes: Some are CEHs and some are not

Five MHP haplotypes contain the markers of a previously reported CEH [3], [4], [6], [21] from *HLA-C* to *HLA-DQB1* (what we define as the “core” or “classical” (for CEH purposes) MHC region). HLA specificities are used in Table 1 to designate each CEH name for historical reasons. The five apparent MHP CEHs are: PGF, the human reference sequence for the MHC, containing the core MHC markers of the CEH “B7,DR15”; COX, representing the CEH “B8,DR3”; QBL, representing the CEH “B18,DR3”; MANN, with the core MHC markers of two “B44,DR7” CEHs (exhibiting both HLA-C*04/C*16 variation and, independently, C4A*0/C4A*3 microvariation); and, DBB, representing the CEH “B57,DR7.”

**Table 1:** MHP cell line and CEH allele-level typing in the core MHC region and *HLA-DPB1*.

		Class I			Complotype				Class II			
Cell Line	Conserved Ext. Haplotype	Number Sequenced	*HLA-C*	*HLA-B*	*CFB*	*C2*	*C4A*	*C4B*	*HLA-DRB1*	*HLA-DQA1*	*HLA-DQB1*	*HLA-DPB1*
**PGF**			*07:02*	*07:02*	UNK^a^	UNK	UNK	UNK	*15:01*	*01:02*	*06:02*	*04:01*
	B7,DR15	23	*07:02*	*07:02*	*S*	*C*	*3*	*1*	*15:01*	*01:02*	*06:02*	*04:01*
	B18,DR15	5	*12:03*	*18:01*	*S*	*Q0*	*4*	*2*	*15:01*	*01:02*	*06:02*	UNK
**COX**			*07:01*	*08:01*	*S*	*C*	*Q0*	*1*	*03:01*	*05:01*	*02:01*	*03:01*
	B8,DR3	30	*07:01*	*08:01*	*S*	*C*	*Q0*	*1*	*03:01*	*05:01*	*02:01*	*01:01*
**QBL**			*05:01*	*18:01*	*F1*	*C*	*3*	*Q0*	*03:01*	*05:01*	*02:01*	*02:02*
	B18,DR3	18	*05:01*	*18:01*	*F1*	*C*	*3*	*Q0*	*03:01*	*05:01*	*02:01*	*02:02*
**MANN**			*16:01*	*44:03*	*F*	UNK	*Q0*	*1*	*07:01*	*02:01*	*02:02*	*02:01*
	C4,B44,DR7	6	*04* b	*44:03*	*F*	*C*	*Q0*/*3*	*1*	*07:01*	*02:01*	*02:02*	UNK
	C16,B44,DR7	4	*16:01*	*44:03*	*F*	*C*	*Q0*/*3*	*1*	*07:01*	*02:01*	*02:02*	UNK
**DBB**			*06:02*	*57:01*	*S*	*C*	*6*	*1*	*07:01*	*02:01*	*03:03*	*04:01*
	B57,DR7	4	*06:02*	*57:01*	*S*	*C*	*6*	*1*	*07:01*	*02:01*	*03:03*	UNK
**MCF**			*03:04*	*15:01*	UNK	UNK	UNK	UNK	*04:01*	*03*	*03:01*	*04:02*
	B44,DR4,DQ7	7	*05:01*	*44:02*	*S*	*C*	*3*	*Q0*/*1*	*04:01*	*03:01*	*03:01*	*04:01*
**SSTO**			*05:01*	*44:02*	UNK	UNK	UNK	UNK	*04:03*	*03:01*	*03:05*	*04:01*
	B49,DR4,DQ8	1	*07:01*	*49:01*	*S*	*C*	*Q0*	*1*	*04:05*	*03:01*	*03:02*	UNK
	B44,DR4,DQ8	6	*05:01*	*44:02*	*S*	*C*	*3*	*Q0*/*1*	*04:01*	*03:01*	*03:02*	*04:01*
	B62,SC33,DR4,DQ8	8	*03:04*	*15:01*	*S*	*C*	*3*	*3*	*04:01*	*03:01*	*03:02*	*04:01*
	B38,SC21,DR4,DQ8	9	*12:03*	*38:01*	*S*	*C*	*2*	*1*	*04:02*	*03:01*	*03:02*	*04:01*
	B60,SC31,DR4,DQ8	10	*03:04*	*40:01*	*S*	*C*	*3*	*1*	*04:04*	*03:01*	*03:02*	*03:01*
	B62,SB42,DR4,DQ8	7	*03:03*	*15:01*	*S*	*B*	*4*	*2*	*04:01*	*03:01*	*03:02*	*04:01*
**APD**			*06:02*	*40:01*	UNK	UNK	UNK	UNK	*13:01*	*01:03*	*06:03*	*04:02*

Shown are MHC alleles for the eight MHP cell lines and, underneath each, for the population CEH(s) that share HLA-DR-DQ specificities with them. Although a known CEH shares HLA-DR-DQ specificities with APD, that CEH does not share significant class II sequence similarity to APD, and is not displayed. Genes are in chromosomal order from telomere to centromere, except *CFB* and *C2* are switched because complotype was historically defined in the order shown. HLA gene alleles are shown at the highest definition known up to 4-digit resolution. Alleles containing “/” indicate microvariation.

a Abbreviations: UNK  =  Unknown (insufficient data).

b This CEH has two possible *HLA-C* alleles: *04:01 and *04:09N [14], [16].

The other three MHP cell lines do not contain a previously described MHC CEH, and it is therefore uncertain how “common” these haplotypes are in any European population. SSTO contains HLA-DQB1*03:05, a DQ8 specificity allele, but is not a reported CEH. Several reported CEHs contain a DR4,DQ8 specificity block (like SSTO) MHC haplotype, although they all have HLA-DQB1*03:02 alleles. The MCF haplotype exists in our database only once and is not a known CEH [21]. The CEH “B44,DR4,DQ7” (in which there is C4B*Q0/C4B*1 microvariation) is the reported CEH most similar to MCF in the class II region. (HLA-DQB1*03:01 is a DQ7 specificity allele.) Finally, the APD MHC haplotype (HLA-C*06:02, B*40:01, DRB1*13:01, DQB1*06:03) does not exist among our 2675 normal Boston haplotypes [21] and has not been reported to be a CEH. In the core MHC region, the CEH [HLA-C*03:04, B*40:01, SC02, DRB1*13:02, DQB1*06:04] (“B60,DR13”) is the reported CEH [3], [4], [6], [21] most similar to the APD haplotype. (HLA-B60 is the specificity of the HLA-B*40:01 allele in this CEH.)

### Haplotype resequencing strategy

**Figure 1:**
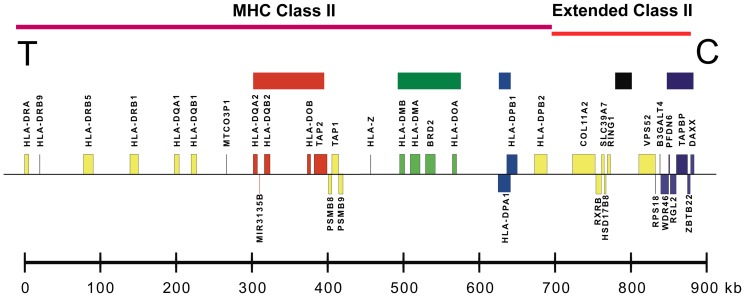
A map of the MHC class II and extended class II regions of chromosome 6p21. Sequenced sub-regions are marked by colored blocks (top). Distances (kb) are to scale from the human reference sequence. Gene locations from *HLA-DRA* on the telomeric (T) end to *DAXX* on the centromeric (C) end are shown.

After aligning MHP sequences [18]–[20], we chose centromeric class II amplicons that would maximize the resultant SNP-DIP haplotypic variation if the MHP sequences were representative of common population haplotypes. We supplemented our initial choices with amplicons to localize transition points where MHP sequences ceased to represent common long-range haplotypes. We designed 56 primer pairs to sequence amplicons in five regions from *HLA-DQA2* to *DAXX* (Figure 1). We resequenced about 27 kb of this 580 kb region. *HLA-DQA2* and *DAXX* are located approximately 80 kb and 660 kb centromeric to *HLA-DQB1*, respectively. The sequenced amplicons and their polymorphisms we report along with their human genome (assembly 37.p10) locations and rs number designations are given in Table S1 and Table S2. Where known polymorphisms are missing in Table S2 from genomic locations within the boundaries of amplicons described in Table S1, all of the haplotypes we report here matched the human reference sequence.

We determined the phased sequences of 158 population haplotypes primarily through segregation analysis in pedigrees. For each pedigree, we determined between two and four founder haplotypes, varying by the number of unrelated haplotypes identifiable in the subjects available for each pedigree. All haplotypes followed Mendelian inheritance patterns except for rare null alleles and intra-family crossovers. For the latter, as with all pedigrees, we only report the unrelated non-crossover founder haplotypes. We achieved 91% to 100% sequence completion in the amplicons from *HLA-DQA2* through *HLA-DMA* and from *RING1* through *DAXX*, 85% sequence completion for the 158 haplotypes in *BRD2* and about 45% to 65% completion from *HLA-DOA* through *HLA-DPB1*. Text S1 provides details about the population haplotypes chosen for each CEH group, and Table S3 provides details of the resequencing coverage (i.e., completion) for each haplotype group and region. Table S5 provides the complete phased SNP-DIP sequence data for all 158 haplotypes and the eight MHP haplotypes for a central portion of the covered region from *HLA-DOB* to *BRD2*. Table S4 provides the MHP and dominant CEH sequences from *HLA-DQA2* to *DAXX*, including the annotated genomic locations of the SNPs and DIPs. The dominant sequences were taken from Table S5 and analogous data from other regions. The CEH groups are organized in numerical order by their HLA-DR/DQ specificities.

### MHP cell line sequence results for regions not previously reported

Some current MHC haplotype scaffolds contain sequence gaps for and/or do not extend centromerically to several of the amplicons we sequenced. We sequenced the QBL (Figure S1), MANN (Figure S2) and DBB (Figure S3) haplotypes within those amplicons. The SNP and DIP alleles within those amplicons for the three MHP haplotypes are highlighted both within the sequences shown in Figures S1–S3 and surrounded by yellow borders in the summary Table S4 and in Table S5. These sequences have assigned GenBank accession numbers as described in the Materials and Methods and the Figures.

#### New QBL sequences

At amplicon DOB1, only 529 nucleotides of this 549 bp region were clearly sequencable due to the homopolymeric region toward the centromeric end, which made the 3′ end of the forward strand unreadable (Figure S1). The 529 nucleotide region of QBL was identical in length to the comparable region of the human reference sequence (PGF), but QBL and PGF differed by one nucleotide at each of the two DIPs (Table S4 and Table S5). However, the two sequences were identical at every SNP. For the other amplicons, all nucleotides between the primers were sequenced. QBL was identical to the human reference sequence in amplicon DOB4. In amplicon DOB5, the QBL sequence differed at the third SNP, but the sequences were otherwise identical.

#### New MANN sequences

At amplicons DOB4 and DOB5, within and 2 kb centromeric to *HLA-DOB*, respectively, MANN (Figure S2) was identical to the QBL sequence, as it was for most of the telomeric region we resequenced. MANN was still representative of its two CEHs within these two amplicons (please see below).

#### New DBB sequences

At amplicon DMP7, only 546 nucleotides of this 599 bp region were clearly sequencable in DBB (Figure S3) due to a homopolymeric region toward the centromeric end, which made the 3′ ends of both strands unreadable. Only 53 of these 546 nucleotides overlapped between the forward and the reverse sequences in DBB. However, of the 546 bp sequenced on either (or both) strand(s), only the fourth SNP differentiated this sequence from the human reference sequence and most other haplotypes (Table S4 and Table S5). For the other two regions, DMP8 and DMP9, all nucleotides between the primers were sequenced, and the forward and reverse readable sequences largely overlapped. At amplicon DMP8, DBB was identical in length to the human reference sequence but differed at the penultimate SNP. In the DMP9 region, the DBB sequence was identical both in length and sequence to the human reference sequence.

### MHP sequence representation of population haplotypes in the class II region

We compared the sequences of the five MHP haplotypes (PGF, COX, QBL, MANN and DBB) containing markers of specific CEHs in the core MHC with population haplotypes bearing the same CEH markers (Table 1). We also compared both the PGF and MANN haplotypes each with a second CEH with which they shared *HLA-DRB1* and *HLA-DQB1* alleles. We compared SSTO and MCF with CEHs that shared at least some class II specificity, allele or sequence identity. Because 90% of the 50 DR4,DQ8 haplotypes we sequenced shared identity to SSTO in the *HLA-DQA2* and *HLA-DQB2* regions, we compared SSTO with the sequences of all six resequenced DR4,DQ8 CEHs (Table 1). We compared the MCF sequence with the only known CEH with which it shares HLA-DR/DQ alleles (please see above). Finally, we compared the APD sequence with the only DR13 population haplotype (out of 13 total) we sequenced having an identical *HLA-DQA2* to *HLA-DOB* sequence. We therefore did not compare the APD sequence with any known CEH because no known CEH shared sequence identity with APD.

**Figure 2:**
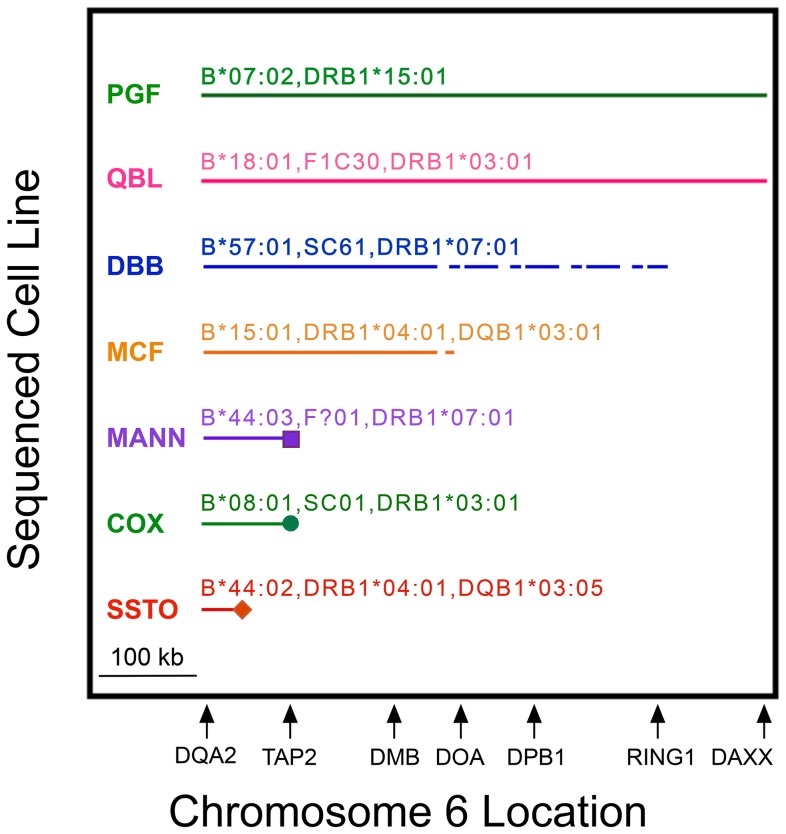
MHP sequences represent CEHs to variable extents in MHC class II from *HLA-DQA2* to *DAXX*. MHP cell line names (left) and their complotypes (when known) and some of their HLA alleles are above their corresponding lines. Shown is the region (solid horizontal line) in which the listed MHP sequence represented the dominant CEH sequence sharing MHC markers identical or similar to the cell line. Cell line specificities or alleles sometimes differed from the “represented” CEHs (see text). A dashed line indicates the region within which a break point between the shared identity of the MHP sequence and the dominant sequence occurred but could not be precisely localized. Icons at the ends of MANN, COX and SSTO show precise break points of shared sequence identity. The relative location of several class II genes (telomere toward centromere from left to right) is shown to scale on the abscissa.

The break point (or lack thereof) at which a MHP sequence no longer represented any CEH is shown in Figure 2. The APD cell line is not shown in Figure 2 because it does not represent a reported CEH. Figure 2 displays were based on analyses of sequences shown in Table S4, and those analyses were based on phased data (Table S5 and analogous data in other regions). PGF had the B7,DR15 CEH dominant sequence and QBL had the B18,DR3 CEH dominant sequence throughout the *HLA-DQA2* to *DAXX* region (Figure 2). PGF also represented the B18,DR15 CEH up to a point within the 1 kb region from intron 8 to intron 6 of *TAP2* (Table S4 and Table S5), where this CEH's dominant sequence began to differ from that of the B7,DR15 CEH.

The other five MHP sequences represented at least one CEH in class II for variable distances. We determined precisely the centromeric break point for the COX sequence. Twenty-five of the 30 B8,DR3-like haplotypes showed sequence identity to COX from *HLA-DQA2* through the DOB9 amplicon within intron 6 of *TAP2*. However, within the same intron, at rs60045856 (SNP ID #147, Table S2), less than 600 bp centromeric to the DOB9 amplicon, the COX sequence differed from all of the previously identical 25 haplotypes (Table S4 and Table S5). The *G* allele at rs60045856 appeared to be a regional tag marker of the B8,DR3 CEH in that all 25 haplotypes identical to the COX sequence up to the DOB9 amplicon possessed this allele whereas all of the other 133 haplotypes reported here (as well as COX and the other seven MHP sequences) had the *T* allele (Table S5). COX never shared significant regional sequence with the dominant B8,DR3 sequence centromeric to *TAP2*.

MANN represented two related B44,DR7 CEHs through at least a region 2 kb centromeric to *HLA-DOB* (amplicon DOB5). Approximately 5.5 kb telomeric to *TAP2*, where the two B44,DR7 CEH dominant sequences diverged, MANN continued to represent the C4,B44,DR7 CEH (despite MANN carrying the HLA-C*16:01-defining allele of the C16,B44,DR7 CEH). Within intron 8 of *TAP2*, the C4,B44,DR7 dominant sequence split into major and minor variants, and MANN ceased to represent either B44,DR7 CEH dominant sequence, although it continued to be identical to the B49,DR4,DQ8 CEH throughout all of the *TAP2* amplicons we sequenced (Table S4 and Table S5). DBB possibly represented the B57,DR7 CEH throughout the class II region in which the CEH maintained a dominant sequence. We could not pinpoint the end of representation by DBB or MCF due to incomplete sequencing (Figure 2; Text S1). However, MCF stopped representing its CEH within the 26.9 kb region at or centromeric to *BRD2* (Table S4), telomeric to lost CEH fixity.

Of the seven MHP sequences in Figure 2, SSTO represented its nearest CEH group for the shortest distance. The centromeric break point for SSTO representation of the CEH HLA-B62,SC33,DR4,DQ8 was within a 13.5 kb region between *HLA-DQB2* and *HLA-DOB*. The SSTO centromeric break point for *any* DR4,DQ8 CEH was narrowed to a different 11.2 kb region between *HLA-DQB2* and *HLA-DOB* (Table S4). However, we can predict the latter break point more precisely using MHP sequence data. Telomeric to rs9276712 (SNP ID #89, amplicon DC13, Table S2), SSTO was identical to the CEHs HLA-B62,SB42,DR4,DQ8, HLA-B60,SC31,DR4,DQ8 and HLA-B38,SC21,DR4,DQ8 and was highly similar to the PGF sequence but differed significantly from the APD sequence. At and centromeric to rs9276712 (for 141 kb, until amplicon DMP1), the SSTO sequence was highly similar to APD but significantly different from PGF (which remained highly similar to the three CEHs). The recombination event that caused this SSTO switch from similarity to PGF to similarity to APD was likely between rs9276712 and rs1158783, the last SNP telomeric to rs9276712 at which the telomeric APD-PGF-SSTO pattern was clear. The distance between the two SNPs is 286 bp in the human reference sequence.

### Only one private mutation was found in a MHP sequence

In an attempt to identify SNP/DIP markers near *TAP2* differentiating the relatively similar sequences of the B18,DR3 CEH (represented by QBL) and the B44,DR7 CEHs (represented by MANN), we resequenced B44,DR7 haplotypes (including MANN) at the DOB7.5 amplicon (Table 2). Among the eight MHP sequences, only MANN had a *T* allele at SNP DOB7.5-2 (rs2857100; ID #2, Table 2). We confirmed this by sequencing MANN at amplicon DOB7.5. However, all nine of the 10 B44,DR7 haplotypes we sequenced (including all five essentially identical to MANN telomeric to the DOB7.5 amplicon) had the *C* allele at rs2857100. We concluded the MANN haplotype had a private mutation at rs2857100. If the *T* allele exists in other B44,DR7 haplotypes, the frequency is likely to be extremely low. This was the only private SNP allele we found in any MHP sequence within a region in which it otherwise had the dominant sequence of a CEH it represented.

**Table 2:** Amplicon DOB7.5 SNPs determined from resequencing.

Amplicon ID	Gene/Region	rs#	Location
DOB7.5-1	*TAP2*	2857101	32,794,676
DOB7.5-2	*TAP2*	2857100	32,794,856

### Dominant sequences of CEHs and their loss of sequence conservation due to recombination in the class II region

**Figure 3:**
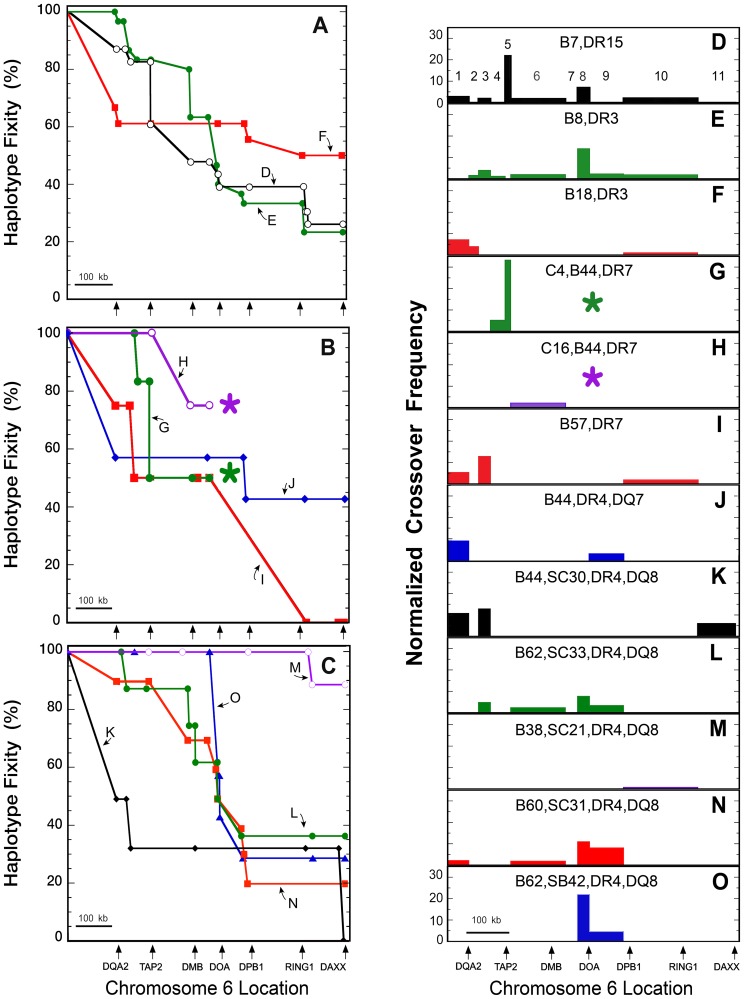
CEH sequence fixity and crossover frequencies from *HLA-DQA2* to *DAXX*. Chromosomal location is shown to scale on the abscissa and starts at the mid-point between *HLA-DRB1 and HLA-DQB1* (A–C) or at *HLA-DQB1* (D–O). The locations of several HLA class II and extended class II genes are marked by arrows below Figures 3A–C and 3O. The 11 regions analyzed for normalized crossover frequency (NCF) are enumerated in Figure 3D. The numbers of haplotypes analyzed for each CEH are given in Table 1. Sequence fixities (A) and NCFs (D–F) are shown for the CEHs B7,DR15 (black open circles), (D); B8,DR3 (green closed circles), (E); and B18,DR3 (red squares), (F). Sequence fixities (B) and NCFs (G–J) are shown for the CEHs C4,B44,DR7 (green closed circles), (G); C16,B44,DR7 (purple open circles), (H); B57,DR7 (red squares), (I); and B44,DR4,DQ7 (blue diamonds), (J). Asterisks (*) in Figures 3B, 3G and 3H indicate that sequence fixities and NCFs could not be determined centromeric to the last data points for the two B44,DR7 CEHs. Sequence fixities (C) and NCFs (K–O) for various DR4,DQ8 CEHs are shown. These include the CEHs B44,SC30/SC31 (black diamonds), (K); B62,SC33 (green closed circles), (L); B38,SC21 (purple open circles), (M); B60,SC31 (red squares), (N); and B62,SB42 (blue triangles), (O). NCFs are normalized to the remaining conserved sequences and to 1 Mb relative to the distance over which crossovers were observed, and values are displayed for 11 sub-regions (Table S2).

From *HLA-DQA2* to *HLA-DQB2*, all CEHs retained a dominant sequence (i.e., maintained “genetic fixity”) shared by 50 to 100% of the population haplotypes in each group (Figure 3A-C). Most CEHs also retained a dominant sequence by *DAXX* (660 kb centromeric to *HLA-DQB1*), although there was usually a gradual reduction in the number of population haplotypes sharing that sequence. CEH dominant sequence results are organized based on the MHP cell line sequences in the order in which they were published. Complete detail of fixity loss for each CEH is provided in Text S1. No results are presented for the DR13,DQ6 CEH because APD did not represent that CEH in the regions we resequenced.

Loss of sequence fixity (Figure 3A–C) is in terms of the gene and/or amplicon at which individual haplotypes stopped sharing SNP/DIP alleles of their CEH dominant sequence. Minor variations, almost always apparently unlinked and isolated private SNPs or DIPs and isolated dominant sequence microvariation, were not counted as loss of sequence fixity (e.g., Table S5). CEH fixity loss is therefore due to past recombination of the dominant sequence with other haplotype sequences. This conclusion is strengthened by our observation that sequences centromeric to the dominant sequence break point are rarely unique and are often found in other CEH groups (e.g., Table S5). We therefore make the explicit assumption that intervening unsequenced regions of population haplotypes sharing a CEH dominant sequence (i.e., telomeric to the break point for any given population haplotype) have similarly limited microvariation and are essentially identical sequences.

Although recombination is the explanation for population haplotype crossover from a CEH dominant sequence (previously identical by descent), it is not possible to calculate CEH recombination rates. The number of meioses experienced by the CEH prior to (or since) its recombination to form any particular population haplotype is unknown. To quantify and display observed population haplotype recombinants responsible for the breakdown of each CEH dominant sequence, we developed a new metric, normalized crossover frequency (NCF, see Materials and Methods ). Our sequencing amplicons and data were not distributed evenly across the region analyzed (Figure 1, Table S1, Table S2, Table S4). We therefore display NCF values, calculated for 11 separate sub-regions (see Materials and Methods and Table S2), on genomic maps drawn to scale (Figure 3D–O). The areas and locations of the bars in those figures quantify and localize the effect of recombination on the loss of CEH fixity displayed in Figure 3A–C.

Sequence recombination is difficult to localize with precision (please see below). Furthermore, the breakdown of a CEH dominant sequence likely varies in different population cohorts. Nevertheless, a few general observations are evident from the results shown in Figure 3. First, the breakdown locations and frequencies vary significantly between different CEHs. Although certain sub-regional crossover sites are more common (e.g., between *HLA-DQB1* and *HLA-DQA2* (sub-region 1), between *HLA-DQB2* and *HLA-DOB* (sub-region 3), between *TAP2* and *HLA-DMA* (sub-region 6), between *BRD2* and *HLA-DPB1* (sub-regions 8 and 9), and between *HLA-DPB1* and *VPS52* (sub-region 10)), none is common to the majority of all analyzed CEHs. Also, some dominant sequences break down gradually in many locations whereas others seem to break down in a more focused fashion. These differences may be due to different relative timelines of CEH expansion and recombination events. Finally, while specific CEHs show a range from high to no sequence conservation through *DAXX*, most CEHs show approximately 50% dominant sequence retention around *BRD2* (in sub-region 7, between *HLA-DMB* and *HLA-DOA*). Below, we highlight the results for specific CEHs.

### Sequence fixity of HLA-DRB1*15:01,-DQB1*06:02 CEHs

The precise location (and, consequently, quantitation) of historic recombination leading to the breakdown of a dominant sequence is often not definable. Perhaps the clearest example of this is the apparent strong crossover frequency for the B7,DR15 CEH in *TAP2* (sub-region 5, Figure 3D). Sequence fixity was maintained for 19 of 23 (83%) B7,DR15 haplotypes from *HLA-DQA2* through intron 8 of *TAP2* (Figure 3A). Beginning in intron 6 of *TAP2*, fixity of the B7,DR15 CEH declined to 14 haplotypes (61%), and was maintained through *TAP2*. The detected crossover of five B7,DR15 haplotypes was to a sequence that defined the B18,DR15 CEH dominant sequence. Thus, the crossovers detected within *TAP2* in these five haplotypes could have occurred anywhere within the region shared by the two CEHs. That region extends telomerically past *HLA-DRB1*. The B18,DR15 CEH dominant sequence from *HLA-DQA2* to *BRD2* (apparently identical to the B7,DR15 dominant sequence through intron 8 of *TAP2*) was found in 80% of the population haplotypes we resequenced (Table S4 and Table S5).

B7,DR15 haplotype resequencing centromeric to *TAP2* showed a gradual loss in CEH fixity through *DAXX* (Figure 3A) and declined below 50% near *HLA-DMB*. The nine B7,DR15 haplotypes identical at *HLA-DPB1* (39% of the original 23) either had the unique exon 2 sequence of or were classically typed as HLA-DPB1*04:01. The dominant B7,DR15 CEH sequence from *HLA-DQA2* through *DAXX* was found in 26% of all resequenced B7,DR15 haplotypes.

### B8,DR3 CEH sequence fixity

All 30 B8,DR3 haplotypes were identical at *HLA-DQA2*. Sequence conservation decreased to 29 haplotypes (97%) at *HLA-DQB2*, to 87% at 16.4 kb centromeric to *HLA-DQB2* (amplicon DC10) and to 25 haplotypes (83%) from *HLA-DOB* through *TAP2* (Figure 3A). B8,DR3 sequence fixity decreased below 50% in the 20 kb region between amplicons DMP10 and DMP11 and declined to 40% at *HLA-DOA* (sub-region 8, Figure 3E). Seven of the 30 haplotypes (23%) had the dominant sequence and were essentially identical through *DAXX*. Six of those contained HLA-DPB1*01:01 (the seventh had HLA-DPB1*03:01). (If the haplotype with HLA-DPB1*03:01 is different from the other six (in spite of having essentially identical SNP-DIP alleles from *HLA-DQA2* to *DAXX*), the dominant sequence at *DAXX* would be represented by only six of 30 (20%) of the studied haplotypes rather than 23%.)

### B18,DR3 CEH sequence fixity

Only 12 of the 18 (67%) B18,DR3 haplotypes showed sequence identity at *HLA-DQA2* (Figure 3A). In contrast to this relatively high crossover frequency between *HLA-DQB1* and *HLA-DQA2* (sub-region 1; Figure 3F), 61% of all 18 haplotypes remained essentially identical to one another and QBL from *HLA-DQB2* through *BRD2*. The only microvariation found among these 11 haplotypes was at the second *BRD2* DIP (ID #204; Table S2). By *DAXX*, nine of the 18 (50%) sequences were still essentially identical.

### Sequence fixity of B44,DR7 CEHs

All 10 B44,DR7 haplotypes shared sequence identity from *HLA-DQA2* through 21 kb telomeric to *HLA-DOB* (amplicon DC13), and 90% of the haplotypes remained essentially identical up to 6.5 kb centromeric to *HLA-DOB* (Figure 3B). As outlined in Text S1, we studied six C4,B44,DR7 (Figure 3G) and four C16,B44,DR7 (Figure 3H) haplotypes. The dominant sequences of these two CEHs became different near and within *TAP2* (Table S4 and Table S5). The DOB6 DIP at 5.5 kb telomeric to *TAP2* and the DOB7-2 SNP 2.8 kb telomeric to *TAP2* defined this split. The four C16,B44,DR7 examples we sequenced maintained sequence identity to one another for the remaining amplicons in *TAP2* and three of these (75%) remained identical through the *BRD2* region. The five essentially identical C4,B44,DR7 haplotypes split into two groups within intron 8 of *TAP2* (at SNP DOB8.4). The dominant sequence, in three of the haplotypes (50%) appeared to be shared through at least 2 kb telomeric to *BRD2*. We did not sequence the haplotypes comprising the dominant sequence of either CEH sufficiently to determine the extent (or lack) of sequence identity centromeric to *BRD2*.

### B57,DR7 CEH sequence fixity

Three of the four (75%) B57,DR7-like haplotypes shared a common sequence from *HLA-DQA2* through approximately 16.5 kb centromeric to *HLA-DQB2*. Sequence fixity declined to two of the four (50%) haplotypes between *HLA-DQB2* and *HLA-DOB* (Figure 3B; sub-region 3, Figure 3I) and continued through *BRD2*. We did not sequence the two identical haplotypes from *HLA-DOA* (amplicon DMP11) through *HLA-DPB1* (amplicon DMP17), but we began sequencing again just centromeric to *RING1* (at amplicon CTB8). At amplicon CTB8, those two haplotypes remained identical to one another. However, 4.5 kb centromeric, at amplicon CTB9, the two haplotypes also differed from one another. Thus, we could only localize the B57,DR7 CEH lost fixity to a 244 kb region between amplicons DMP10 and CTB9 (Figure 2 and Figure 3B).

### B44,DR4,DQ7 CEH sequence fixity

Although there was a significant loss of CEH sequence fixity between *HLA-DQB1* and *HLA-DQA2*, four of the seven (57%) B44,DR4,DQ7 haplotypes retained identical sequence from within intron 1 of *HLA-DQA2* through at least *HLA-DOA* (Figure 3B and Figure 3J). Within the first intron of *HLA-DPA1* (amplicon DMP15), the number of identical CEH sequences decreased to three haplotypes (43%). The haplotype that became different was not sequenced at amplicon DMP14. The three identical haplotypes retained sequence identity to one another through *DAXX*. The sequence presented in Table S4 contains HLA-DPB1*04:01 and represents the dominant B44,DR4,DQ7 CEH sequence.

### Sequence fixity of DR4,DQ8 CEHs

#### Class II fixity of DR4,DQ8 CEHs

Of the 50 sequenced DR4,DQ8 haplotypes, 41 represented six CEHs (Table 1). Forty-five of these were essentially identical to the SSTO sequence from *HLA-DQA2* through *HLA-DQB2* (amplicon DC9; Table S4). Four of five non-SSTO sequences had a different common sequence from *HLA-DQA2* to 16.4 kb centromeric to *HLA-DQB2* (amplicon DC10). Three of the four (75%) comprised half the examples we sequenced of the B44,DR4,DQ8 CEH. The fifth non-SSTO sequence was the sole example of the B49,DR4,DQ8 CEH.

The 45 DR4,DQ8 haplotypes identical at *HLA-DQA2* and *HLA-DQB2* included 33 of the 34 (97%) haplotypes comprising the other four CEHs. These CEHs shared identical sequence both in that region and in a 45 kb region from *HLA-DMB* through *BRD2* (boxed region in Table S4). However, the dominant sequences of these four major DR4,DQ8 CEHs differed from one another within the intervening 170 kb.

#### B49,DR4,DQ8

This haplotype differed from all other DR4,DQ8 haplotypes at and centromeric to *HLA-DQA2* (Table S4). Whether this single sequence represents the CEH is unknown at this time.

#### B44,DR4,DQ8

Of six examples with the markers of this CEH, two had the SC30 complotype and four had SC31. *C4B* microvariation did not correlate with differences among the six haplotypes at or centromeric to *HLA-DQA2*. The B44,DR4,DQ8 CEH had two equally representative centromeric class II sequences and was the least fixed CEH (Figure 3). From *HLA-DQA2* to *HLA-DOB*, three of the six haplotypes (50%) remained identical to one another (and most other DR4,DQ8 haplotypes). Just 2 kb centromeric to *HLA-DOB*, one sequence became different. Within intron 9 of *TAP2* (at amplicon DOB9), the remaining two haplotypes lost sequence identity to one another.

The other three B44,DR4,DQ8 haplotypes (50%) shared a different sequence from *HLA-DQA2* through amplicon DC10, identical to DBB. At amplicon DC13 and centromeric through *ZBTB22*, two of the three haplotypes had a unique common sequence that differed from DBB. Thus, two of the original six B44,DR4,DQ8 haplotypes (33%), both of which were typed as HLA-DPB1*04:01, maintained a fixed and identical sequence through *ZBTB22*, approximately 650 kb centromeric to *HLA-DQB1* (Figure 3C and Figure 3K, Table S4).

#### B62,SC33,DR4,DQ8

Sequence fixity for this CEH declined steadily, and three (38%) retained the dominant sequence of this CEH through *DAXX* (Figure 3C and Figure 3L).

#### B38,SC21,DR4,DQ8

This CEH had the highest fixity at *DAXX* of all the CEHs we studied (Figure 3C and Figure 3M), and eight haplotypes (88%) remained identical to one another through *DAXX*.

#### B60,SC31,DR4,DQ8

Sequence conservation in this CEH declined steadily (Figure 3C and Figure 3N). *HLA-DPB1* typing and centromeric sequence analysis suggested that fixity decreased to 20% at that locus and continued centromeric to *DAXX*.

#### B62,SB42,DR4,DQ8

From *HLA-DQA2*, all seven examples of this CEH retained a unique dominant sequence through *BRD2*. From *HLA-DOA* to *HLA-DPB1*, the fixity of this CEH declined steadily to 29% (Figure 3C and Figure 3O). One of those two fixed haplotypes was typed as HLA-DPB1*04:01.

### Sequence fixity of DR13,DQ6 haplotypes

Although APD did not represent any known CEH in the resequenced region, APD shared sequence identity with its single represented population haplotype from at least *HLA-DQA2* through *TAP2*, a distance of almost 100 kb. The previously identical sequence differed from the APD sequence at every SNP 2.1 kb telomeric to *HLA-DMB* but was otherwise identical at amplicons DMP2 through DMP6. Within and near *HLA-DMB* and *HLA-DMA* (amplicons DMP1-6), no other haplotype we sequenced had the APD sequence, and only two other haplotypes (both non-standard haplotypes from other groups) had the same sequence as the DR13,DQ6 haplotype we report in Table S4. The APD sequence in amplicons DMP7 through DMP13 has not been reported, but its sequence near and in the HLA-DP genes differed from the other DR13,DQ6 haplotype we report.

## Discussion

Identifying the genetic elements responsible for complex genetic diseases requires knowing the genomic haplotype architecture of the population(s) in which the diseases exist. Toward that goal, the MHP made a major advance in the early to mid-2000's by determining the sequences of eight European Caucasian MHC haplotypes [17]–[20]. However, although the MHP sequences were described as “common” European Caucasian MHC haplotypes, that remains an open question [4]. Other than a comparison of the eight sequences with 180 European Caucasian population haplotypes at 54 SNPs covering 214 kb of the MHC class II region (from *HLA-DRB9* to 20 kb centromeric to *HLA-DQB1* (Figure 1)) [20], no systematic study has determined the representative nature of each MHP haplotype's complete sequence.

MHC CEHs are common population haplotypes [1]–[6], [8]–[16]. Outside of their core conserved region from *HLA-C* to *HLA-DQB1*, CEHs contain dominant alleles on the telomeric class I side at *HLA-E* [14] and *HLA-A* [1]–[6], [8]–[11], which is consistent with the region between *HLA-A* and *HLA-C* being one of notably low recombination [22]. The class II region between *HLA-DQB1* and *DAXX* is thought to be less conserved than that analogous distal class I region, and class II contains several reported recombination hotspots [22], [23] and regional LD breaks [24] (which are thought to be related). Furthermore, the best-characterized CEH, [HLA-B8, SC01, DR3], previously showed significant variability centromeric to *HLA-DQB1* [9], [10], [25]. By contrast, previous reports also documented dominant *HLA-DPB1* alleles for a number of European Caucasian CEHs [3], [4], [15], including the B8,DR3 (in shortened nomenclature) CEH.

We used an amplicon resequencing approach [10] to determine the dominant class II sequences centromeric to *HLA-DQB1* and to delineate the breakdown of sequence conservation among multiple examples of previously identified CEHs sharing telomeric class II alleles or specificities with the eight MHP sequences. The dominant class II sequences were unique for each CEH, and we confirmed or discovered over 300 SNP and DIP markers. The phased polymorphisms of each CEH dominant sequence are shown in Table S4. Most CEHs showed significant sequence conservation (“fixity”) centromeric to *HLA-DQB1*, crossing multiple reported recombination hotspots [22], [23]. Although a few CEHs lost a dominant sequence by *DAXX* (660 kb centromeric to *HLA-DQB1*), most CEHs retained a dominant sequence throughout the region. Seven of the eight MHP sequences represented the dominant class II sequence of at least one CEH for varying distances.

Several general observations derive from our data. First, microvariation was low within a CEH's dominant class II sequence, even at DIPs, similar to findings within the core MHC for the two CEHs previously reported [9]–[11]. We detected only a single private mutation among the MHP sequences within regions where they otherwise represented the dominant sequences. These findings suggest CEH sequences are recent enough not to have sustained significant mutation during their expansion. Second, CEH dominant sequence conservation appears to be lost primarily due to recombination events with other relatively high frequency haplotypes because non-consensus sequences centromeric to the point of differentiation typically are identical to other common regional sequences. These are often the local fragments of other CEH dominant sequences. Third, except in a few cases where CEHs split apart from a common sequence shared by related CEHs, the dominant sequences did not usually transition to multiple examples of a conserved minor sequence past the recombination point. Although this last observation remains to be confirmed in studies of larger numbers of the same CEH, it also suggests the minor variant recombinants are relatively recent compared to the ages of the original CEHs.

A fourth conclusion is somewhat complex. Although the location of dominant sequence breakdown varied between CEHs (Figure 3D–O) and did not appear to be primarily at previously reported recombination hotspots or LD breaks [22]–[24], many CEHs showed a steady loss of fixity throughout the region. The major reported recombination hotspots in the region are [22]–[24]: a) between *HLA-DQB1* and *HLA-DQA2* (near *MTCO3P1*) [22]; b) within intron 2 of *TAP2* (just centromeric to the most centromeric amplicon of *TAP2* we sequenced) [22]; c) just telomeric to *HLA-DMB*, near the next amplicon we sequenced centromeric to *TAP2* [23]; d) between *BRD2* and *HLA-DOA* [22], [23]; e) between *HLA-DOA* and *HLA-DPA1* [22], [24]; and, f) between *HLA-DPB1* and *RING1* [24]. However, losses of sequence conservation occurred in the region between *HLA-DQA2* and intron 2 of *TAP2*, a region not previously reported to contain a recombination hotspot.

Our directly observed haplotype results reveal complexity missed by a casual analysis of LD maps. The results we present regarding CEH structure renew questions we previously raised regarding both LD and recombination hotspots [3]. However, our study was not designed to identify nor to challenge the existence of recombination hotspots in the extended class II region, and further study of this region is warranted.

**Figure 4:**
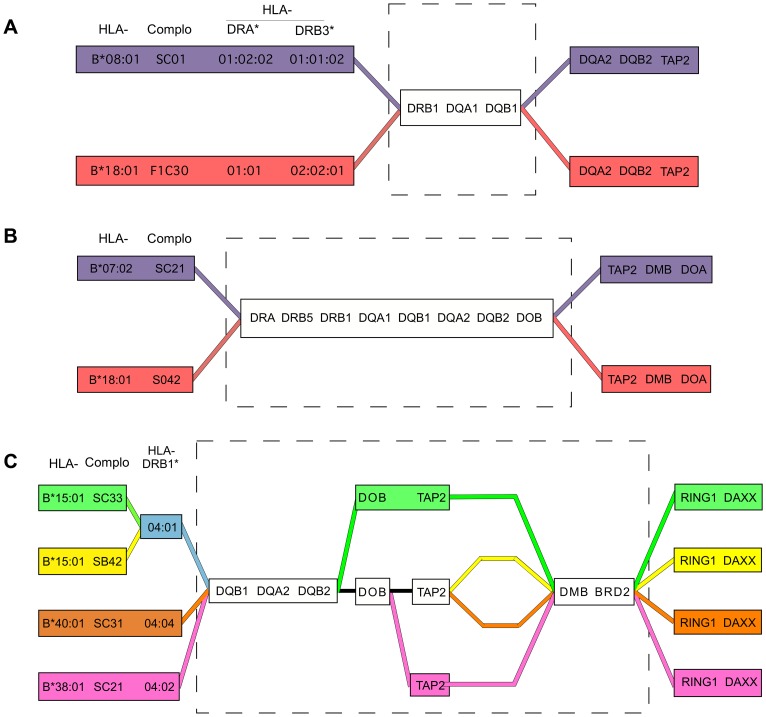
Shared and divergent sequences in related CEHs. A) A region of nearly identical sequence for the B8,DR3 and B18,DR3 CEHs was previously reported [19] and is represented by the broken line rectangle, and ends just centromeric to *MTC30P1*, approximately 50 kb centromeric to *HLA-DQB1* [18]. B) Shared sequence for the B7,DR15 and B18,DR15 CEHs is shown in the broken line rectangle. Sequence identity for these two CEHs ends centromerically between introns 8 and 6 of *TAP2*. C) Shared and divergent sequences for four DR4,DQ8 CEHs are shown in the broken line rectangle. HLA-B*15:01 and HLA-B*40:01 are alleles of the B62 and B60 specificities, respectively.

An interesting feature of several CEH pairs and groups is a pattern of shared sequence identity surrounded both telomerically and centromerically by regions in which the CEHs differ significantly (Figure 4). This “block” nature of CEHs and haplotype groups sharing regional alleles has been noted previously [3]–[8], [13]–[16], [19], [22], [25]. The MHP reported [19] a 158 kb “SNP desert” from *HLA-DRB1* and *MTCO3P1* between the two DR3 CEHs (Figure 4A). Our study expands upon that concept and provides a richer picture of these relationships.

For example, the B7,DR15 and B18,DR15 CEHs were previously known to share alleles within the HLA-DR/DQ block [1], [3]–[6], [8], but it was unknown whether they had identical or distinct sequences centromeric to *HLA-DQB1*. Our results show these two CEHs share sequence identity throughout the 88 kb stretch from *HLA-DQA2* through intron 8 of *TAP2*, centromeric to which they maintain fixed but distinctly different sequences (Figure 4B). Although the two CEHs theoretically could have different sequences between *HLA-DQB1* and *HLA-DQA2* and in the domains we skipped within the 88 kb region mentioned above, previously published results suggest such variation would be minimal. The MHP showed, using a set of dense SNP typings, that a set of (HLA-DRB1*15:01, -DQB1*06:02) population haplotypes were identical to one another centromeric to *HLA-DQB1* until they split into primarily two subtypes in a region near or within *TAP2* [19]. The sudden *TAP2* transition they reported was likely *both* the centromeric break point of the shared sequence for the two DR15 CEHs and the continuation of the two separate but conserved CEH sequences we report here. Similarly, the two B44,DR7 CEHs [16] may share essential sequence identity for the region from *HLA-B* to *HLA-DOB* but have separate conserved sequences on either side of that larger than 1.5 Mb region. The two CEHs may have recombined in the early history of a common ancestral haplotype and expanded separately.

We observed a more complex structural pattern among the DR4,DQ8 CEHs than among the DR15 CEHs: two separate regions of shared sequence separated by a variable region of sequence divergence. Specifically, four DR4,DQ8 CEHs telomerically identical at *HLA-DQB1*, *HLA-DQA2* and *HLA-DQB2* and centromerically identical from *HLA-DMB* through *BRD2*, each had different sequences for varying distances within the 170 kb span between the two sub-regions (Figure 4C). This pattern may be analogous to the pattern within the core MHC region exhibited by the related CEHs [HLA-B62, SB42, DR4, DQ8] and [HLA-B62, SC33, DR4, DQ8] (which, interestingly, are the most divergent of the four DR4,DQ8 CEHs within the 170 kb mentioned above). These patterns of alternating blocks of shared and divergent sequence/alleles may be a type of CEH supergroup microvariation created by early differentiation from a common ancestral sequence due to recombination or, perhaps more likely, localized hypermutation followed by expansion of separate but related CEHs.

Although our dense sequencing results raise questions specific to the class II region, the main issue is essentially the same question we and others have asked about CEHs generally: How can long-range conserved sequences comprise up to half a population's haplotypes crossing numerous putative recombination hotspots or regions of LD breakdown? For example, one of the strongest reported MHC recombination hotspots is located in the *TNF*-*LTA* region [22], yet that region is located within the core MHC, the only human genomic region well-documented to contain CEHs.

We conclude CEHs are recent expansions of separate ancestral progenitors. Thus, multiple population examples of each CEH are essentially identical by descent but have spread through the population into pedigrees that are not now highly related. The few mutations within a stretch of conserved sequence can be used to calculate the age of the long-range haplotype [10], [26]. However, plausible values for the variables in such calculations are often difficult to verify.

We also conclude LD values are not particularly useful indicators of population haplotype architecture [3], [4]. LD variation is likely useful to demarcate localized changes in the relationships between individual haplotypes, but LD is all too often simplistically and incorrectly interpreted to suggest the population haplotype architectual dominance of short blocks of conserved sequence separated by narrow regions of relatively frequent randomized sorting. It is likely not coincidental that the MHC is both the region most often studied by segregation analysis in pedigrees and the only well-documented region to contain megabase-length CEHs.

Haplotype sequence and population haplotype architecture accuracy requires both direct observation and the consideration of long-range sequence fixity. Whole genome sequencing will soon allow direct determination of full haplotype sequences if analyzed appropriately [27]. This requires either sequencing individual chromosomes after physical isolation [28] or sequencing moderate to large pedigrees to phase pedigree data directly [29], [30]. The latter allows both sequence integrity crosschecking and directly observed recombination. Samples homozygous for a particular long-range haplotype are useful for identifying putative CEH alleles [10], [17]–[20], [31], [32], but such cell lines are rare. Direct haplotype determination and counting [2] is the only method capable of revealing the details of haplotype structure and population haplotype architecture essential for disease gene localization [4]. Computational phasing to “impute” haplotype structure in unrelated subjects has been advocated for monetary or feasibility reasons, but this does not usually provide accurate haplotype structure [33].

Reports over 30 years show that MHC CEHs are high population frequency (“common”) megabase-length conserved sequences [1], [3]–[6], [8]–[16], [23], [24]. The evidence for CEH sequence conservation (with minor microvariation) increased whenever loci were defined at higher resolution or at intervening locations. We update and improve the definition of the centromeric points up to which the published reference MHC sequences essentially represent CEH dominant sequences. The dominant class II CEH sequences we provide (far from a complete list) should be useful for future European Caucasian haplotype comparisons. More complete resequencing of larger numbers of pedigree-determined haplotypes is required to determine population haplotype architecture both within the MHC and throughout the genome. Furthermore, non-European CEHs [34] must be studied in a similar manner. Finally, an appreciation of long-range haplotype sequence conservation throughout the genome is required to localize efficiently the genomic structural elements responsible for complex genetic traits (including disease susceptibility).

## Materials and Methods

### Ethics statement

All participants gave informed consent in accordance with Institutional Review Board (IRB)-approved protocols. All work was conducted under IRB protocols approved by the Immune Disease Institute (or its predecessors) and/or Boston Children's Hospital IRBs.

### Human subjects

Blood samples were provided by 180 individuals in 43 unrelated families and by 10 unrelated subjects (the latter homozygous for portions of the MHC), mostly from the Boston metropolitan area. We obtained extensive demographic and personal health information (including family histories) from all subjects. The relatively diverse European Caucasian population in Boston and our recruitment methods make it highly unlikely any of the pedigrees or unrelated subjects are directly related to one another. We also obtained B-lymphocytic cell lines of 15 individuals in four families from the Human Biological Data Interchange (HBDI; Philadelphia, PA). International Histocompatibility Workshop (IHW) homozygous cell lines (n = 12), including three of the MHP (DBB, MANN and QBL), were used for a limited number of haplotypes. All samples had been typed at classical markers within the MHC prior to selection, although typing was conducted at various resolutions (from serological to high resolution DNA typing). Pedigrees were chosen to obtain multiple examples of a wide variety of MHC CEHs or at least the HLA-DR/DQ fragments of CEHs putatively represented by MHP haplotypes. *HLA-DPB1* typing was not considered during subject and haplotype selection so that the degree of fixity in the centromeric class II region was random.

### Sample extraction

DNA was extracted from EDTA-treated blood, peripheral blood mononuclear cells or B-lymphocytic cell lines. Genomic DNA was isolated using the QIAmp DNA mini kit (Qiagen, Valencia, CA).

### MHC Typing

Molecular MHC allele typing was performed by PCR and sequence-specific oligonucleotide probes (in-house or Lifecodes) or by sequence-specific primer kits (Invitrogen) at low to high resolution. Some HLA types were identified serologically [35]. *CFB* (previously known as *BF*) and *C4* allele typing was by agarose gel electrophoresis and immunofixation with specific antisera; *C2* alleles were determined by isoelectric focusing of serum in polyacrylamide gels and a C2-sensitive hemolytic overlay [36]. MHC complement gene haplotypes or complotypes are designated by their *CFB*, *C2*, *C4A*, and *C4B* alleles, in that arbitrary order [7]. Null or *Q0* alleles are simply designated 0. Thus, FC31 indicates the complotype CFB*F, C2*C, C4A*3, C4B*1. Complotypes for some IHW cell lines were described previously [30], [31]. IHW cell typing was known (http://www.ebi.ac.uk/ipd/imgt/hla/cell_query.html) and/or verified as described above.

### Alignment of MHP sequences to develop a resequencing strategy

We analyzed eight different MHC class II and extended class II sequences determined by the Sanger Institute [17]–[20] for distinguishing SNPs and deletion/insertion polymorphisms (DIPs). Currently available MHC sequence data for these cell lines may be found via: http://www.ucl.ac.uk/cancer/medical-genomics/mhc or http://www.ensembl.org/index.html or at the URL listed under “MHC Typing.” MHP haplotypes represent the human reference sequence (PGF) as well as the following alternative sequences for the human MHC: ALT_REF_LOCI_1 (APD), ALT_REF_LOCI_2 (COX), ALT_REF_LOCI_3 (DBB), ALT_REF_LOCI_4 (MANN), ALT_REF_LOCI_5 (MCF), ALT_REF_LOCI_6 (QBL), and ALT_REF_LOCI_7 (SSTO).

We used an amplicon-based resequencing approach [10] to distinguish the dominant sequences of CEHs in the class II region. CLC Combined Workbench software program (CLCBio LLC, Cambridge, MA) was used to align these sequences for the region from *MTCO3P1* to *DAXX* (Figure 1). After aligning all MHP sequence data available for the eight haplotypes, we analyzed the entire region from *MTCO3P1* to *DAXX* to find an optimal distribution of amplicons that balanced the needs for relatively even coverage and for maximizing differences between the sequences. After preliminary resequencing and localization of regions in which some of the MHP haplotypes appeared to cease representing many population haplotypes or in which we had poor sequencing results, we added or substituted amplicons. Finally, in some cases, we skipped relatively large regions having known low polymorphism.

### DNA sequencing

We designed primers, using a version of Primer 3 software (http://frodo.wi.mit.edu), at monomorphic (or near-monomorphic) positions in regions near or within genes that would likely offer maximal differentiation of the various MHP haplotypes. The primer sequences we used and the amplicons we resequenced are shown in Table S1. We sequenced a total of approximately 27 kb using 56 sets of primers covering five separate regions spanning a total distance of approximately 580 kb of genomic DNA. In some cases where sequence phase could be determined without some members of a particular pedigree, the DNA of those members was not sequenced, but we often sequenced all members of a pedigree to confirm results for a particular haplotype in multiple carriers of that haplotype. We also sequenced portions of three MHP cell lines in data gaps of current scaffolds, and we report and have provided that information to GenBank.

PCR products were excised from agarose gels and purified using the QIAEX II gel extraction kit (Qiagen) or were drawn out of recovery wells directly (Lonza, Inc.) and sequenced by dideoxy sequencing using Big Dye Terminator V3.0 chemistry (Genewiz, Inc., South Plainfield, NJ and/or Davis Sequencing, Inc., Davis, CA). All sequences were analyzed and compared using both alignment software and direct visual inspection of chromatograms. DIP sizes in heterozygotes were usually decipherable and deducible in both directions based on the known sequence surrounding the DIP. At least two individuals inspected visually and agreed upon the sequence of each chromatogram used to determine sequence.

Excluding private mutations, we identified 274 SNPs and DIPs in the 342 kb region from *HLA-DQA2* to *HLA-DPB1* and 34 SNPs and DIPs in the 103 kb region from centromeric to *RING1* to *DAXX* (Table S2). We defined the centromeric point where a particular MHP sequence no longer represented a haplotype group as the location at which the dominant sequence shared by those haplotypes began to contain SNP and DIP alleles not in the MHP sequence.

### Haplotype assignment

The vast majority of haplotypes (*n* = 132; 83.5%) were phased by segregation analysis in pedigrees, showed Mendelian inheritance patterns (except in rare cases of null alleles or detected crossovers) and were assigned unique identifiers as unrelated founder chromosomes. Six unrelated subjects or IHW cell lines each known not to be consanguineous were homozygous for specific haplotypes throughout the region analyzed and provided 12 additional unrelated chromosomes (7.6%). Six IHW cell lines either known to be consanguineous or of unknown status provided six additional unrelated chromosomes (3.8%). Finally, four unrelated subjects known not to be consanguineous who were heterozygous for at least some portion of the region studied provided the final eight unrelated chromosomes (5.1%).

Haplotype phasing in the classical CEH region (between *HLA-C* and *HLA-DQB1*) was established for 95% of all haplotypes or was inferred from known CEH allele combinations. Over 96% of SNP and DIP alleles were unambiguously phased: a) by segregation analysis in pedigrees [1], [2]; or, b) using IHW or locally-identified MHC homozygous samples. Such cell lines were assumed to be of consanguineous origin unless known not to be and received only one haplotype assignment. The remaining alleles (<4% overall and <4% in all regions except for *HLA-DPB1*, where the percentage of inferred phasing was 10.8%) were assigned to haplotypes by inference as follows. In a family in which all subjects were heterozygous identical at a locus or in a heterozygous individual without relatives, one of the alleles was arbitrarily assigned to one of the haplotypes to be consistent with its surrounding (unambiguous) markers, defined by the unambiguous haplotypes in the group to which it belonged or, if the haplotype was no longer representative, by all unambiguous haplotypes. Phasing of the remaining pedigree haplotype(s) was/were thus established. We report here on 158 haplotypes (and an additional seven at *HLA-DQA2*) that fell into one of the eight MHP groups.

### Mapping, gene annotation and variation analysis

Physical distances between MHC genes, locations and amplicons were found at the NCBI website (http://www.ncbi.nlm.nih.gov/projects/genome/guide/human/index.shtml). We used the human reference sequence NC_000006.11 from Genome Reference Consortium assembly GRCh37.p10 and reference sequence (rs) numbers are from dbSNP build 138. All novel SNP (*n* = 1) and DIP (*n* = 7) variations (shown in Table S2) were submitted to dbSNP (http://www.ncbi.nlm.nih.gov/snp/) using the handle CAALPER. All novel DNA sequences for the three MHP cell lines have GenBank accession numbers (http://www.ncbi.nlm.nih.gov/genbank/) KF880997-KF880999 (for QBL), KF881000-KF881006 (for MANN) and KF881007-KF881009 (for DBB) (Figures S1–S3). To determine sequence fixity, we assumed sequence identity within intervening regions we did not resequence among the population haplotypes bearing the genotypic markers and/or dominant sequence of a CEH (except for rare private mutations and infrequent microvariations).

### Normalized crossover frequency

To quantify and represent crossover events leading to the breakdown of CEH dominant sequences, we define a new metric: normalized crossover frequency (NCF). NCF is the fraction of remaining dominant sequences of a single CEH that begin to differ from the dominant sequence due to apparent recombination within a defined region, normalized over a unit (1 Mb) distance. Our data were not distributed evenly across the region we studied, and we therefore calculated our data over sub-regions of varying size (Table S2). Thus, we required normalization to a unit distance to compare the sequence breakdown by separate crossovers. We displayed these data in a bar graph format in which the abscissa is drawn to genomic scale. Therefore, the areas (*not* the heights) of the bars representing NCFs are compared to determine the relative contribution of regional recombinants to the breakdown of CEH sequence conservation. NCF was calculated using the equation:

NCF  =  (crossovers/total remaining haplotypes) × (1 Mb/distance covered)

where:

a) “crossovers” are the number of haplotypes that lost the CEH dominant sequence centromeric to the prior (telomeric) analyzed region due to recombination events (as opposed to minor microvariation in an otherwise identical sequence), and include both any crossovers directly observed in the currently analyzed region and any deduced to have occurred between the currently and prior analyzed regions.

b) the “total remaining haplotypes” are the number of remaining population haplotypes having the dominant sequence of a given CEH throughout the region immediately telomeric to the analyzed region. For the first region (*HLA-DQA2*), it was assumed all population haplotypes of a given CEH had the dominant sequence at the centromeric end of *HLA-DQB1*.

c) the “distance covered” is measured by subtracting the genomic position of the most centromeric point of the prior region (the region immediately telomeric to the currently analyzed region) from the genomic position of the most centromeric point of the currently analyzed region.

As an example, if 16 population haplotypes of a single CEH had the dominant sequence through *HLA-DQA2* and 3 of these crossed over to a non-dominant sequence by the centromeric end of the *HLA-DQB2* region (in which the distance from the most centromeric polymorphism analyzed in the *HLA-DQA2* region through the most centromeric polymorphism analyzed in the *HLA-DQB2* region is 21,096 bases), the NCF would be:

(3/16) × (1,000,000/21,096)  = 8.9

and the “total remaining haplotypes” for the next region (between *HLA-DQB2* and *HLA-DOB*, covering 25,927 bases) would be 13 (i.e., 16–3).

## Supporting Information

Figure S1 Sequence data for the **QBL** cell line in the *HLA-DOB* region. The QBL cell line was sequenced in three regions not previously reported. Data are for the positive strand, reading from the telomere toward the centromere, and the chromosome 6 human genome location (relative to the human reference sequence NC_000006.11 GRCh37.p10 assembly) of the telomeric and centromeric bases are given. Polymorphisms (SNPs and DIPs) are shown in bold with a gray background (with DIPs double-underlined). Sequence data were obtained from both strands except where the sequence is single-underlined. GenBank accession numbers are shown for each sequence.
(DOC)

Figure S2 Sequence data for the **MANN** cell line in the *HLA-DOB* and *WDR46* to *DAXX* regions. MANN was sequenced in seven regions not previously reported. Data are for the positive strand, reading from the telomere toward the centromere, and the human chromosome 6 location (relative to the NC_000006.11 GRCh37.p10 assembly) of the telomeric and centromeric bases are given. Polymorphisms (SNPs and DIPs) are shown in bold with a gray background (with DIPs double-underlined). Sequence data were obtained from both strands except where the sequence is single-underlined. GenBank accession numbers are shown for each sequence.
(DOC)

Figure S3 Sequence data for the **DBB** cell line in the *BRD2* region. DBB was sequenced in three regions not previously reported. Data are for the positive strand, reading from the telomere toward the centromere, and the human chromosome 6 location (relative to the NC_000006.11 GRCh37.p10 assembly) of the telomeric and centromeric bases are given. Polymorphisms (SNPs and DIPs) are shown in bold with a gray background (with DIPs double-underlined). Sequence data were obtained from both strands except where the sequence is single-underlined. GenBank accession numbers are shown for each sequence.
(DOC)

Table S1 Resequencing primers, amplification product sizes and resequencing distance covered. Forward and reverse primer sequences for all amplicons reported in this study are provided along with the amplicon PCR product size and the distance sequenced within each amplicon. The telomeric and centromeric boundary positions of the sequenced regions are provided for each amplicon based on the human reference sequence NC_000006.11 GRCh37.p10 assembly.
(XLS)

Table S2 Non-private polymorphisms determined from resequencing. The SNPs and DIPs within the amplicons sequenced are provided, including their local name, their reference sequence (rs) number, their chromosomal position in the human reference sequence (based on the NC_000006.11 GRCh37.p10 assembly) and the amplicon name within which they were sequenced. Eleven sub-regions for which NCF values were determined are shown on the right.
(XLS)

Table S3 Resequencing completion for haplotypes by group and class II region. Provides detailed information on the percentage of completion for haplotype resequencing organized by haplotype group, genetic region and partial and complete sequencing.
(XLS)

Table S4 MHP cell line and dominant CEH/class II fragment haplotype sequences from *HLA-DQA2* to *DAXX*. Not all polymorphisms are displayed: within the boundaries of the sequenced amplicons, cell line and CEH/dominant population haplotype sequences contained the allele present in the human reference sequence if not shown. Allele data for the eight cell lines sequenced by the MHC Haplotype Project (data available from: http://www.ucl.ac.uk/cancer/medical-genomics/mhc) are shown along with the dominant sequence(s) we determined for related CEHs.
(XLS)

Table S5 MHP cell line and 158 population haplotype polymorphism sequences from telomeric to *HLA-DOB* to *BRD2*. Within the boundaries of the sequenced amplicons, MHP and population haplotype sequences contained alleles in the human reference sequence if not shown. Annotated locations have chromosome 6 positions shown in Table S2.
(XLS)

Text S1 Supplementary Results text provides more detailed information on the regions where MHP sequences represented European CEHs and on CEH fixity throughout the centromeric MHC class II and extended MHC class II regions.
(DOC)
