# Supplementary material for: Dominant Sequences of Human Major Histocompatibility Complex Conserved Extended Haplotypes from HLA-DQA2 to DAXX
Source: PLoS Genet. 2014 Oct 9;10(10):e1004637. doi: 10.1371/journal.pgen.1004637 (PMC4191933; doi:10.1371/journal.pgen.1004637)
Supplement: Figure S1 — Sequence data for the QBL cell line in the HLA-DOB region. The QBL cell line was sequenced in three regions not previously reported. Data are for the positive strand, reading from the telomere toward the centromere, and the chromosome 6 human genome location (relative to the human reference sequence NC_000006.11 GRCh37.p10 assembly) of the telomeric and centromeric bases are given. Polymorphisms (SNPs and DIPs) are shown in bold with a gray background (with DIPs double-underlined). Sequence data were obtained from both strands except where the sequence is single-underlined. GenBank accession numbers are shown for each sequence. (DOC) [file pgen.1004637.s001.doc]

Sequence data for the **QBL** cell line in the *HLA-DOB* region

**AMPLICON DOB1 (Chromosome 6 location: 32767979 to 32768507):**

GenBank accession number: KF880997

cagcgtcaggtgagagtgttataccacatatcactagcccagagaaagactgacatttcaaatttgaagtacatttcttccagaatgtgtactgctttcacgccatcacagctgaacaatcttaagtggaaccatc**a**taagtcaggaaccatctgtatttgatgttttaaaccatgtcatgtataacttttt**t**aaaaaaaagataaaataattttaaatattaaaataggaaaattttaaaaa**g**aaaaaagacattttt**tc**ttttctttttcttttctttttttttttttt**tta**ggcagagtcttg**c**tctgtcacccaggctggagtgcagtg**a**c**a**tgatcttggctcactgca**g**gctccacctcctgggttcacaccattctcctgcctcagcctccc**g**tgtagctgggactacaggcacctaccaccatgcccagctaatttttcatattttttagtagagacagggtttcactgtgttagccaggatggtct**c**gatctcctgacct**c**gtgatctgctcacctcagc

**AMPLICON DOB4 (Chromosome 6 location: 32782340 to 32783102):**

GenBank accession number: KF880998

gtggggtcctctctgggtacactgtcacctctggttgcactaggaag**g**gaggaaaaatgagacaccgtgaaagaaaaccaccaagctgggacaggagattctttagggactatcactatgtctaatctctttcccagatcacccaagtgaacacaaagtataggcaagtctcagcccccaagatcagtaacagggtatgtcaatgcctgtcaggaggatttagactttctgaggtactccca**c**aattactgcttctctttgaggg**c**acaatagccctcgaagtccctgagaaccttgggggtctgagaccaagatcacagtggctgacttgtgaggataatatatcacagctggggccagaacatctacacagacaaccatttatcctaaagcagaaaattgcttgtaagaaagaagagccatggccaggttcacatgggggacattcctgagcc**c**cgccagacctcagcttccagctcaccttttctccccacagtgaagggtgcgcccagcctgtagttgtgtctacagaccccatccacggcctgtctgctcctctccaagagatccagccggctgttccactgctcagcatctggctgccccagcttggtcaatgccacaaacatccccacatcactgtcgaaacgtacatactcctccaagttaaagatgaatctgaccacaaactgcaccttttctgtcccgttggtgaagtaacagtcagcctttgcctgaatcacaaa**a**tcttctggaaaaccaa

**AMPLICON DOB5 (Chromosome 6 location: 32786605 to 32786939):**

GenBank accession number: KF880999

agcttggcacttttgcccttctgccttcctccttgtgaggactgttacactggaatgatttgactcaagtgtttagttaagtattctttcagtaaaacctagacagtaaaacactatctttaagcaaataaaaccaaaagtgcaaattgtaattcaccatctatgttattattatttaaagggcaatgtttactcatcatttcacatcatctttcagcatgaaatgtg**c**ccctgatttgcctatactgtgcatgttaagaatgaacccagggtatca**t**ggtaaccacaagttcacttcagtgacttttttca**a**gtcgatggccaaggcatcaaat
