# Supplementary material for: Dominant Sequences of Human Major Histocompatibility Complex Conserved Extended Haplotypes from HLA-DQA2 to DAXX
Source: PLoS Genet. 2014 Oct 9;10(10):e1004637. doi: 10.1371/journal.pgen.1004637 (PMC4191933; doi:10.1371/journal.pgen.1004637)
Supplement: Figure S2 — Sequence data for the MANN cell line in the HLA-DOB and WDR46 to DAXX regions. MANN was sequenced in seven regions not previously reported. Data are for the positive strand, reading from the telomere toward the centromere, and the human chromosome 6 location (relative to the NC_000006.11 GRCh37.p10 assembly) of the telomeric and centromeric bases are given. Polymorphisms (SNPs and DIPs) are shown in bold with a gray background (with DIPs double-underlined). Sequence data were obtained from both strands except where the sequence is single-underlined. GenBank accession numbers are shown for each sequence. (DOC) [file pgen.1004637.s002.doc]

Sequence data for the **MANN** cell line in the *HLA-DOB* and *WDR46* to *DAXX* regions

**AMPLICON DOB4 (Chromosome 6 location: 32782340 to 32783102):**

GenBank accession number: KF881000

gtggggtcctctctgggtacactgtcacctctggttgcactaggaag**g**gaggaaaaatgagacaccgtgaaagaaaaccaccaagctgggacaggagattctttagggactatcactatgtctaatctctttcccagatcacccaagtgaacacaaagtataggcaagtctcagcccccaagatcagtaacagggtatgtcaatgcctgtcaggaggatttagactttctgaggtactccca**c**aattactgcttctctttgaggg**c**acaatagccctcgaagtccctgagaaccttgggggtctgagaccaagatcacagtggctgacttgtgaggataatatatcacagctggggccagaacatctacacagacaaccatttatcctaaagcagaaaattgcttgtaagaaagaagagccatggccaggttcacatgggggacattcctgagcc**c**cgccagacctcagcttccagctcaccttttctccccacagtgaagggtgcgcccagcctgtagttgtgtctacagaccccatccacggcctgtctgctcctctccaagagatccagccggctgttccactgctcagcatctggctgccccagcttggtcaatgccacaaacatccccacatcactgtcgaaacgtacatactcctccaagttaaagatgaatctgaccacaaactgcaccttttctgtcccgttggtgaagtaacagtcagcctttgcctgaatcacaaa**a**tcttctggaaaaccaa

**AMPLICON DOB5 (Chromosome 6 location: 32786605 to 32786939):**

GenBank accession number: KF881001

agcttggcacttttgcccttctgccttcctccttgtgaggactgttacactggaatgatttgactcaagtgtttagttaagtattctttcagtaaaacctagacagtaaaacactatctttaagcaaataaaaccaaaagtgcaaattgtaattcaccatctatgttattattatttaaagggcaatgtttactcatcatttcacatcatctttcagcatgaaatgtg**c**ccctgatttgcctatactgtgcatgttaagaatgaacccagggtatca**t**ggtaaccacaagttcacttcagtgacttttttca**a**gtcgatggccaaggcatcaaat

**AMPLICON CTB14 (Chromosome 6 location: 33256025 to 33256681):**

GenBank accession number: KF881002

gttaggatggcagtaaagcttcccaatatgaagcaagtataccaacataaaaacgagaaaagacagtccca**t**aaggcagggaatgggggtccagattagggctcactcacccaggttcttcagcaagcagcagctcagaacgagcagctttgatacttgtttcctcttcctcagcttcagccacctcaagtcggcttcgagttttggctttagaatgtggtagctgtaacattgttggtggggaggagtggcagaagaaccacaggataagtggggtcacaggagagctacctgtcccagcctccatccaactcacccagacactccctgcctccagcacttcccaactctccggctggacctcacctttcg**g**gatttgtcaatgcgacagaacttctggaccacttccacagggacgggggcggggcctgggaatggatcttggg**c**ctaggggaaaggaggacgcaattagcagacagccttggattgaccccaaccctctcactctc**a**aggaacgaggcggcctgcctcgccacccatcaggtcccacgctcaccccggacaagccgcgctgggactccgggttcttccattctcggggtttcttcgggacctgaggcttcttagagatccgagacttctttaagatgtaagcat

**AMPLICON CTB15 (Chromosome 6 location: 33275334 to 33275707):**

GenBank accession number: KF881003

gtgcccagctaatttttgtatttttggtagagatggggtttcaccatgttgcccaggctggtcttgaactcccgacctcaggtgatcc**g**cctgctttggtctcccaaagtgctgggattacaggcatgagccaccacgcctggcctctagctctgcttcttacacactgtgtgtccttgggcaaattatttaactggtttgtgtcctatatttatccatatgcaatacagggataatattaaaacctacaacctatggttgttgagaggaataagtgagattatgcatataaagtgcttagaacagggcctggcatatagaaaatacttgataaatgttagctgttactattttcattaccttcatcactatca

**AMPLICON CTB16 (Chromosome 6 location: 33281763 to 33282361):**

GenBank accession number: KF881004

agtcgcggggttcgctcacccaaagccacagcgaggagcagagacagggacttcatggcgctgcgacctcctcagccat**tt**agcctcctcttcctcctttcactttcactttcctccaaagggcggcatgaggggcggtggaaatccccgctctggttaggtgaaggtgcctgggggaccggtgtttccccactggccaggcagggacccgggtagatcctctccagttctcaccagga**t**accccagccttaccgcgccctcctggactacccagcagccccgagttcgagccctccccaaccccaggccctcccccgccccccaactcctgtgtgtgctctccaacatccacttgcccgaaaaccattactccggcttccccctatctgtgccgcgtccccagcaaacacacgggtt**g**tcgggaagccaagtaaatgaccaataaatattttaatcactgttaaaaaaaataaaaaccttgtactcctacgacttactccctccttgtctccacccactcctccatgagaaccgagttgggaatttccacgggaagtcgggggtggcggggaga**g**acagggtagaaataaagagcgca

**AMPLICON CTB17B (Chromosome 6 location: 33285104 to 33285369):**

GenBank accession number: KF881005

atctacctttttggtgttttgcacccactttttgggagggggcagggcagctctgctactgaaaaccaacgcttgctccatctcccctcaggctatgccccccaagctctctcgccgaccacgccccctttcgccccagcttctctagccccgcccct**t**tccaggccca**ccccccccc**gtgccccgcccactatcgggcctttcgaccccgccccttgtctacctccgcccacaacggaccccgcccccccccgctccgccccaagcg

**AMPLICON CTB18A (Chromosome 6 location: 33290590 to 33291214):**

GenBank accession number: KF881006

ctctatatccccgcccccgcctctgatccccgcaccgtccggcccccacctcagaaaccgtctctcgaggcgaccctcgcc**g**caattctcagaacctcgcatggttccctccgccttccttcccactcccaccgcaggccccactacggaccggaagtcacagagtttccgccttcatgcaactaagcgccgccatattgtcgtacggaacacaggcttcctgtggccggaggtg**g**cagtaggcccgccccgcgaacacctccagtgcggcccacatagtca**a**cggtctcttccaggtcggagtttgtct**c**cccgaacccaggcgtcccaaagcagctgggggcc**g**ccattttgccgtacggcactggctacgcccggactccggtgcgcagccagtggagctcttttcacccggtgcttctacgactccgccaatcagaaacttcctgcctggggcccaaccgccggagagtagcgcgtagggaggaccgagcctcgtttccaaggaggggcaggggaaccgaacagggtggattaggaattgggctttccaaagctgtgcagagtttcagggaagggcagaagtctcttaaaagggaagtaaaaccttttctttcagttgggc
