# Supplementary material for: Dominant Sequences of Human Major Histocompatibility Complex Conserved Extended Haplotypes from HLA-DQA2 to DAXX
Source: PLoS Genet. 2014 Oct 9;10(10):e1004637. doi: 10.1371/journal.pgen.1004637 (PMC4191933; doi:10.1371/journal.pgen.1004637)
Supplement: Figure S3 — Sequence data for the DBB cell line in the BRD2 region. DBB was sequenced in three regions not previously reported. Data are for the positive strand, reading from the telomere toward the centromere, and the human chromosome 6 location (relative to the NC_000006.11 GRCh37.p10 assembly) of the telomeric and centromeric bases are given. Polymorphisms (SNPs and DIPs) are shown in bold with a gray background (with DIPs double-underlined). Sequence data were obtained from both strands except where the sequence is single-underlined. GenBank accession numbers are shown for each sequence. (DOC) [file pgen.1004637.s003.doc]

Sequence data for the **DBB** cell line in the *BRD2* region

**AMPLICON DMP7 (Chromosome 6 location: 32936151 to 32936696):**

GenBank accession number: KF881007

aggctaccccgctaggccgcgggtagtgggggagggg**g**cgctgaggcaggaggtcagcacccgggcgcgggctcccgccccacgaaa**a**gcgcgcgctccaagccccgccgc**c**ggagatgcggttccggtccggacgcctgcgcactacggctctccccgcagcctctggccctccttccccctcccccagtcagggcgcacccttgcgcctgcgctgtgtgt**a**ttcctggtctgcggcagccatgctgaactcgtatggagaggcgagtgggggggacagagtccaggac**t**gcgggataggaagctggggatatggacaagcagcagcgttatagcgctctgggtttcgggacataggcctgggccatgcggcccccttggccccttggcgcgacccccaggaacgttcggaaagctggtcctcgtggctgggggaaaggcggggggtgggggggaagcgggcacgtgaccccggtcagccaatctgggtgctgctgacgtggccgcgcggccccgatgctctccccacccccccagcccgttcgg

**AMPLICON DMP8 (Chromosome 6 location: 32937812 to 32938365):**

GenBank accession number: KF881008

gattttccggaatgcagggaataaacgagagcaatgtctggctgcccttttcctaaggcctagtattttctcagcctcctaagttttta**t**tccatggccggccccctgatgggcctctgtcctggcctgcagagccccggtggagaaaagcagatttgggaggttgggcc**g**ctagggggaggggaaaaggcctctgca**a**agttgctgtgtcattgccctccatgctgcagccaccca**a**acggggccgcttgtacttttgggg**g**ccagggcctgatccctg**g**ctgggggaaggggactctgctctcctgacgctcattttcccccgccctcccggggtttgccctactcggggggtcagaagacaggagattggcggccattttagac**g**cagtaaccgaggttggagttgaagggctactgcagaggagggagggtggcgtggttgcagc**t**caaggacctaggcccttacgagcccttcc**c**gggcgagggg**ga**atctta**c**cgtatatttgttcacctacgttgattatttttcccagatacgtacacaagtttgt

**AMPLICON DMP9 (Chromosome 6 location: 32943110 to 32943507):**

GenBank accession number: KF881009

cctataagcatttatttttctgtggttctgac**c**taacattt**t**tttatttaggattatcacaaaattataaaacagcctatggacatgggtactattaagaggagacttgaaaacaattattattgggctgcttcagagtgtatgcaagattttaataccatgttcaccaactgttacatttacaacaaggtgagtttttctgtgtgttcatttagtaggtggggagaaacagtaa**c**ttctattattgctggatatgttgtctacataaagtttaaatcctttgctactgaaggtgtt**a**tccaggtagggtagtcggagtcttaaaaacctgactctagatggtactattgaacacagtgatgtgacttcagagctctagttgaaggttatttagaaca
