# Supplementary material for: Dominant Sequences of Human Major Histocompatibility Complex Conserved Extended Haplotypes from HLA-DQA2 to DAXX
Source: PLoS Genet. 2014 Oct 9;10(10):e1004637. doi: 10.1371/journal.pgen.1004637 (PMC4191933; doi:10.1371/journal.pgen.1004637)
Supplement: Text S1 — Supplementary Results text provides more detailed information on the regions where MHP sequences represented European CEHs and on CEH fixity throughout the centromeric MHC class II and extended MHC class II regions. (DOC) [file pgen.1004637.s009.doc]

**Supplementary Results**

We present a detailed analysis of the dominant sequences and loss of fixity in CEHs as resequencing proceeded centromerically from *HLA-DQA2* to *DAXX* and our findings as to the representative nature of the MHP cell line sequences in comparison with the population CEHs they putatively model. The results are organized within the MHP group meant to represent that/those CEH(s). Unless stated otherwise, when individual haplotypes ceased to maintain the dominant group sequence, the centromeric sequence did not comprise a subset of the group. Such haplotypes also did not usually break away from the group in large numbers at a single point. Generally, the only exceptions were: a) where large distances (e.g., 100 kb) separated adjacent amplicons; or, b) when related CEHs separated into two groups (e.g., the B18,DR15 CEH breaking away from the B7,DR15 CEH or the points at which various DR4,DQ8 CEHs broke away from the main group).

**Sequence fixity of *HLA-DRB1*15:01,-DQB1*06:02* CEHs**

We sequenced 30 (*HLA-DRB1*15, DQB1*06*) haplotypes, 23 of which also contained at least one class I marker and, when typed, at least three of the complotype markers of the [*HLA-C*07:02, B*07:02, SC31, DRB1*15:01, DQB1*06:02*] (“B7,DR15”) CEH. (HLA-DR15 is a group specificity that contains *HLA-DRB1*15:01*.) Five other haplotypes contained at least one class I marker and at least three complotype markers of the CEH [*HLA-C*12:03, B*18:01, S042, DRB1*15:01, DQB1*06:02*] (“B18,DR15”). Twenty-three of the 30 (77%) haplotypes showed sequence identity to PGF from *HLA-DQA2* through intron 8 of *TAP2*, including four of the five (80%) B18,DR15 haplotypes and 19 of the 23 (83%) B7,DR15 haplotypes. Thus, the B7,DR15 and B18,DR15 CEHs had identical sequence at all 122 SNPs and 13 DIPs in the 9474 bp we resequenced in that 87.9 kb region. This represents an 11% sample of the chromosomal sequence from the first intron of *HLA-DQA2* through intron 8 of *TAP2*.

Beginning in intron 6 of *TAP2*, only 14 of the 23 (61%) previously identical haplotypes had sequence identity to PGF. All of these 14 were B7,DR15 haplotypes. The other nine previously identical class II haplotypes had a different (but common) sequence. Four of the five (80%) B18,DR15 haplotypes (all previously identical to the B7,DR15 CEH) were among these nine haplotypes, whereas only five of 23 (22%) B7,DR15 haplotypes showed the same different sequence. Thus, 14 (61% of the original 23) B7,DR15 haplotypes continued to show sequence identity to PGF through all of the resequenced *TAP2* regions (Table S5). However, the B18,DR15 CEH was no longer represented by the PGF sequence at some point within the region from intron 8 to intron 6 of *TAP2*. The break point of shared sequence was therefore pinpointed to within 1 kb. All four of the B18,DR15 examples that had retained a dominant sequence at this split retained sequence identity to one another through *BRD2* (Table S5). We will describe in a later report the alleles of the dominant B18,DR15 CEH sequence centromeric to *BRD2*, which remained fixed through at least *DAXX*.

The telomeric border of the next region we resequenced was almost 100 kb centromeric to *TAP2* and just telomeric to *HLA-DMB* (Figure 1). Of the 14 B7,DR15 haplotypes with identical sequence by *TAP2*, 11 (79%) had identical sequence in the 10 amplicons we resequenced in and around *HLA-DMB*, *HLA-DMA* and *BRD2* (containing at least 45 SNPs and two DIPs for a cumulative total of 4.9 kb resequenced of a region with a total length of 48.2 kb). Thus, 11 of the original 23 B7,DR15 haplotypes (48%) remained fixed and identical to PGF through the 3’ UTR of *BRD2*.

Resequencing of an additional seven amplicons in the *HLA-DOA* to *HLA-DPB1* region (with at least 65 SNPs and four DIPs) showed that nine of the 14 previously identical haplotypes (64%) to PGF continued to be represented by PGF at least through exon 2 and the telomeric end of intron 2 of *HLA-DPB1*. (We did not resequence one of the nine haplotypes in the DMP17 amplicon covering the second exon of *HLA-DPB1*, but this haplotype had been classically typed as *HLA-DPB1*04:01*, the same allele as all of the other six identical haplotypes that had been typed at that locus.) These nine haplotypes comprised 39% of the original B7,DR15 haplotype group.

Of the nine B7,DR15 haplotypes identical to the PGF sequence through *HLA-DPB1*, seven remained identical to PGF through amplicon CTB11 (17.5 kb centromeric to *RING1*) and six (26% of the original 23) remained identical to PGF through the most centromeric point we resequenced, the 5’ UTR of *DAXX*. Thus, the PGF sequence represented the most common B7,DR15 haplotype sequence from *HLA-DQA2* through *DAXX*, comprising 26% of all the B7,DR15 haplotypes we resequenced.

**B8,DR3 CEH sequence fixity**

We sequenced 30 (*HLA-DRB1*03, DQB1*02*) haplotypes, 27 of which contained at least one class I marker and, if typed, at least three of the complotype markers of the CEH [*HLA-C*07:01, B*08:01, SC01, DRB1*03:01, DQB1*02:01*] (“B8,DR3”). The other three haplotypes were followed in this CEH group (and not the B18,DR3 CEH represented by QBL) because their *HLA-DQA2* sequence was identical to the COX sequence. All 30 haplotypes (100%) were identical to the COX sequence at *HLA-DQA2*. Sequence identity to COX slightly decreased to 29 haplotypes (97%) at *HLA-DQB2*, to 87% at 16.4 kb centromeric to *HLA-DQB2* (amplicon DC10) and to 83% from 12 kb telomeric to *HLA-DOB* (amplicon DOB1) through intron 6 of *TAP2* (amplicon DOB9).

Within *TAP2* intron 6, at amplicon DOB10, less than 400 bp centromeric to DOB9, the COX sequence differed from the dominant B8,DR3 sequence (still 83% of all population haplotypes) at a single SNP, rs60045856 (ID #147, Table S2). The *G* allele at this SNP appears to be a tag marker of the B8,DR3 CEH: all 25 DR3 haplotypes identical to the COX sequence up to amplicon DOB9 possessed this allele whereas the remaining 142 haplotypes reported here (including COX and the other seven MHP sequences) possessed the *T* allele at rs60045856. The COX sequence also differed from all of the dominant 25 B8,DR3 haplotypes (which remained identical to one another) at a DIP 3.6 kb centromeric to rs60045856 in intron 3 of *TAP2* (rs9280197; ID #157, Table S2). Thus, the centromeric break point where the COX sequence no longer represented the B8,DR3 CEH was pinpointed to a region of less than 500 bp within intron 6 of *TAP2*. COX also differed from the common B8,DR3 CEH sequence at 10 of 23 SNPs at the next centromeric region we resequenced (in and around *HLA-DMB* and *HLA-DMA*). Indeed, COX never shared any significant regional sequence with the B8,DR3 CEH dominant sequence or any other CEH or major class II group we studied centromeric to its breakpoint with the B8,DR3 group.

Just telomeric to *HLA-DMB*, 24 of the previously identical haplotypes (80% of all 30 haplotypes) retained sequence identity through the DMP1 primer set and 19 (63%) retained sequence identity from *HLA-DMB* (amplicon DMP2) through at least the DMP10 primer set (within the 3’ UTR of *BRD2*). Therefore, the dominant B8,DR3 sequence (not COX) represented 63% of the B8,DR3 haplotypes through *BRD2* (Table S5). This fixity declined to 40% at *HLA-DOA* and to 33% at *HLA-DPB1*. Only seven of the original 30 haplotypes (23%) had the dominant sequence and remained essentially identical to one another by *DAXX*. Six (86%) of these were typed as *HLA-DPB1*01:01*.

**B18,DR3 CEH sequence fixity**

We sequenced 18 (*HLA-DRB1*03*, *DQB1*02*) haplotypes, 16 of which contained the complotype (F1C30) and, in some cases, at least one class I marker of the CEH [*HLA-C*05:01, B*18:01, F1C30, DRB1*03:01, DQB1*02:01*] (“B18,DR3”). The two haplotypes lacking F1C30 were followed in this group because their *HLA-DQA2* sequences were identical to QBL. Only 12 of the 18 (67%) haplotypes showed sequence identity to QBL at *HLA-DQA2*. Half of the other six haplotypes showed *HLA-DQA2* sequence identity to MCF (please see below), and the other three haplotypes were each different from one another. In contrast to this relative lack of fixity at *HLA-DQA2* (compared with most of the other groups we studied), 11 of the 12 identical haplotypes at *HLA-DQA2* (61% of all 18 haplotypes) remained essentially identical to one another and QBL from *HLA-DQB2* through *BRD2*. The only microvariation found among these 11 haplotypes was at the second *BRD2* DIP (ID #204; Table S2, Table S5). By *DAXX*, nine of the 18 haplotype sequences (50%) we followed in this group were identical to QBL. QBL was one of only two MHP sequences representative of the dominant sequence in its group through *DAXX* (Figure 2).

**Sequence fixity of B44,DR7 CEHs**

We sequenced 10 (*HLA-DRB1*07, DQB1*02*) haplotypes, nine of which also contained the complotype FC31 or FC01 and/or at least one class I marker of the highly related CEHs *[HLA-C*04, B*44:03, FC0/31, DRB1*07:01, DQB1*02:02*] (“C4,B44,DR7,” that has microvariation at *HLA-C* as *HLA-C*04:01* or *HLA-C*04:09N*) and [*HLA-C*16:01, B*44:03, FC0/31, DRB1*07:01, DQB1*02:02*] (“C16,B44,DR7”) (in both of which the *C4A* locus exhibits allele microvariation). Two haplotypes were not typed at *HLA-C*. Both were assigned to the *HLA-C*04* CEH group: one because its centromeric sequence was of that group and the other by randomized assignment. The single haplotype lacking either the complotype or class I markers of these CEHs was also followed in the *HLA-C*04* group because its *HLA-DQA2* sequence was identical to MANN (which contains a “G” allele at rs28371353 (ID #12, Table S2) that appears to be unique to this CEH) and its centromeric sequence was of the *HLA-C*04* group. Thus, of the 10 studied haplotypes, six were assigned to the *HLA-C*04* group and four were assigned to the *HLA-C*16* group. Only two of five haplotypes typed at *HLA-DPB1* shared an allele (*HLA-DPB1*04:01*; although one each was in the two different B44,DR7 CEHs), which was different from that in MANN (*HLA-DPB1*02:01:02*).

All 10 haplotypes showed sequence identity to MANN at *HLA-DQA2*, which continued through a region 21 kb telomeric to *HLA-DOB* (amplicon DC13). One haplotype (assigned to the *HLA-C*04* group) was different within the *HLA-DOB* region, but 90% of the haplotypes remained essentially identical to MANN up to 2 kb centromeric to *HLA-DOB* (amplicon DOB5). (One haplotype had three SNP differences from the others, but otherwise retained the common dominant sequence centromeric to *HLA-DOB*.) Within the DOB6 and DOB7 amplicons, the nine common sequences split into two separate groups distinguished by a 5T/6T variation at the DOB6 DIP and a *G*/*T* variation at SNP DOB7-2. All four of the C16,B44,DR7 examples were 5T-*T* and four of the five C4,B44,DR7 examples were 6T-*G* (Table S5). Within intron 8 of *TAP2*, the C4,B44,DR7 split into major (three examples) and minor (two examples) groups. The minor group had the same sequence as MANN. This was the most centromeric region we sequenced in which the MANN sequence was identical to any B44,DR7 CEH sequence. The centromeric boundary of the MANN sequence representation of the minor C4,B44,DR7 CEH sequence ended somewhere within the 97 kb region from intron 2 of *TAP2* (SNP ID #159, Table S2) to 2 kb telomeric to *HLA-DMB* (SNP ID #160).

The four C16,B44,DR7 population haplotypes maintained sequence identity to one another for the remaining amplicons we sequenced in *TAP2*, and three of these haplotypes (75%) remained identical through the *BRD2* region. The dominant C4,B44,DR7 CEH sequence, in three of the haplotypes (50%), appeared to be shared through at least 2 kb telomeric to *BRD2* (Table S5). We did not sequence the haplotypes comprising the dominant sequence of either CEH sufficiently to determine the extent (or lack) of sequence identity centromeric to *BRD2*.

**B57,DR7 CEH sequence fixity**

We sequenced four *HLA-DRB1*07* haplotypes containing the SC61 complotype of the CEH [*HLA-C*06:02, B*57:01, SC61, DRB1*07:01, DQB1*03:03*] (“B57,DR7”). Three of these haplotypes also contained *HLA-B*57*. Three of the four (75%) haplotypes shared a common sequence with DBB from *HLA-DQA2* through approximately 16.5 kb centromeric to *HLA-DQB2* (amplicon DC10). Starting only 4.5 kb centromeric to DC10 (at amplicon DC13) and continuing through *BRD2*, the DBB sequence was shared with only two of the four (50%) haplotypes. This dominant B57,DR7 sequence from *HLA-DQA2* through *BRD2* had numerous unique (“tag”) alleles (Table S4). We did not sequence the two haplotypes identical to DBB from DMP11 (*HLA-DOA* region) through DMP17 (*HLA-DPB1*), but took up sequencing again at CTB8 (just centromeric to *RING1*). By CTB8, the two previously identical haplotypes were identical to one another but differed from the DBB sequence. However, in the next amplicon (CTB9), 4.5 kb centromeric to CTB8, the two haplotypes also differed from one another. Thus, we could only localize the point at which the B57,DR7 CEH broke apart in terms of fixity below 50% to a 244 kb region between amplicons DMP10 and CTB9. The region within which DBB represented this dominant B57,DR7 CEH sequence could not be pinpointed much better: it was between amplicons DMP10 and CTB8.

**B44,DR4,DQ7 CEH sequence fixity**

We sequenced 15 (*HLA-DRB1*04, DQB1*03*) haplotypes with a *DQB1* allele typed at low to high resolution as of the DQ7 specificity ("DR4,DQ7" haplotypes). All of the haplotypes we typed to at least 4-digit resolution (*n*=12) bore *HLA-DQB1*03:01*. Of the 11 DQ7 haplotypes we typed at *HLA-DRB1* to 4-digit resolution, eight were *HLA-DRB1*04:01*, two were *HLA-DRB1*04:07* and one was *HLA-DRB1*04:05*. The CEH [*HLA-C*05:01 B*44:02, SC30/1, DRB1*04:01, DQB1*03:01*] (“B44,DR4,DQ7”) is the previously reported CEH most similar to the MCF haplotype. Six of the DR4,DQ7 haplotypes we sequenced bore the markers of that CEH. One haplotype was not typed at *HLA-B* but otherwise had the CEH markers. Thus, we categorized seven of the 15 (47%) DR4,DQ7 haplotypes as examples of the CEH.

Eight of the 15 (53%) DR4,DQ7 haplotypes, including four of the seven (57%) B44,DR4,DQ7 haplotypes, shared an identical sequence with MCF within the *HLA-DQA2* first intron. None of the other seven haplotypes shared a common sequence from *HLA-DQA2* through *TAP2*, and therefore we concluded a second major variant of the DR4,DQ7 group did not exist. At *HLA-DQB2*, sequence identity dropped to seven of the 15 (47%) DR4,DQ7 haplotypes (but still 57% of the B44,DR4,DQ7 haplotypes). (That haplotype, number 188 (Table S5), differed from the dominant sequence at several SNPs within three amplicons in and immediately centromeric to *HLA-DQB2* but retained the dominant sequence centromeric to the DC8 amplicon. This was a unique occurrence. We counted it as a crossover, but show its dominant sequence retention in Table S5.) An additional non-CEH haplotype became different 2.5 kb centromeric to *HLA-DQB2* (at amplicon DC8). However, sequence identity to MCF was maintained from that point centromerically to at least intron 3 of *BRD2* (amplicon DMP9) in six of the 15 (40%) DR4,DQ7 haplotypes, which still included four of the seven (57%) B44,DR4,DQ7 haplotypes. Within exon 12 and the 3’ UTR of *BRD2* (DMP10), another non-B44,DR4,DQ7 haplotype became different from the others, bringing fixity of all DR4,DQ7 haplotypes to 33% (five of 15) but still four of seven (57%) for the CEH. The MCF sequence within exon 12 and the 3’ UTR of *BRD2* has not been reported and we did not sequence it. Therefore, we could not determine whether the sequence was representative of the B44,DR4,DQ7 CEH within that region.

Within amplicon DMP11, 4.1 kb telomeric to *HLA-DOA*, MCF had a different sequence from the five previously identical haplotypes. Those haplotypes, which still included three B44,DR4,DQ7 haplotypes, remained identical to one another through at least exon 4 of *HLA-DOA* (amplicon DMP13). We concluded the MCF sequence ceased to represent the B44,DR4,DQ7 CEH somewhere within the 26.9 kb region between the centromeric end of amplicon DMP9 and the SNP DMP11-2.

Within the first intron of *HLA-DPA1* (amplicon DMP15), the number of identical sequences decreased to four of the original 15 (27%) DR4,DQ7 haplotypes and three of the original seven (43%) B44,DR4,DQ7 CEHs. The haplotype that became different was not sequenced at amplicon DMP14. The remaining four identical haplotypes retained sequence identity to one another through *DAXX*. Three of those four haplotypes had been typed at *HLA-DPB1*, and all three contained *HLA-DPB1*04:01*. Therefore, the sequence presented in Table S4 contains *HLA-DPB1*04:01* and represents the dominant B44,DR4,DQ7 CEH sequence.

**Sequence fixity of DR4,DQ8 CEHs**

We sequenced 47 (*HLA-DRB1*04*, *DQB1*03*) haplotypes in which *HLA-DQB1* was typed at serological or low to high resolution as of the DQ8 specificity. We name these "DR4,DQ8 haplotypes." We also included in this group one haplotype typed serologically as HLA-DR4,DQ3 and two haplotypes not typed at *HLA-DQB1* because their *HLA-DQA2* sequences were identical to 90% of the other DR4,DQ8 haplotypes, bringing the total number of haplotypes to 50. The SSTO haplotype (*HLA-C*05:01, B*44:02, DRB1*04:03*) contains *HLA-DQB1*03:05*, which is a member of the DQ8 sub-group, but neither this allele nor this haplotype was in our database. The CEH [*HLA-C*05:01, B*44:02, SC30/1, DRB1*04:01, DQB1*03:02*] (“B44,DR4,DQ8”; in which there is *C4B*0*/**1* microvariation) is the CEH in our database most similar to the SSTO MHC haplotype. All DQ8 haplotypes we typed to 4-digit resolution (*n*=30) contained *HLA-DQB1*03:02*.

We sequenced many HLA-DR4,DQ8 haplotypes because this class II specificity block is shared by several CEHs. We followed six of these CEHs (Table S4) among 41 of the 50 haplotypes (82%) to determine which were represented well in the class II region by SSTO. The remaining 9 haplotypes were a variety of non-CEH DR4,DQ8 haplotypes. At the 67 polymorphisms from the *HLA-DQA2* first intron to approximately 2.6 kb centromeric to *HLA-DQB2* (amplicon DC9), 45 of the 50 (90%) DR4,DQ8 haplotypes were identical to SSTO (except for a single private SNP in one haplotype). Four of the five non-SSTO sequences had a different common sequence from the *HLA-DQA2* first intron to 16.4 kb centromeric to *HLA-DQB2* (amplicon DC10). These haplotypes were enriched (three of four; 75%) in the B44,DR4,DQ8 CEH mentioned above. The fifth non-SSTO sequence was the sole example of the CEH [*HLA-C*07, B49, SC01, DRB1*04:05, DQB1*03:02*] ("B49,DR4,DQ8"; please see below).

Within amplicon DC10, eight of the 45 (18%) common DR4,DQ8 haplotypes, seven of which had the alleles of the CEH [*HLA-C*03:04, B*15:01, SC33, DRB1*04:01, DQB1*03:02*] (“B62,SC33,DR4,DQ8”; please see below), began to have a common sequence different from that of SSTO. Thus, SSTO no longer represented the consensus sequence of the B62,SC33,DR4,DQ8 CEH, where it began to differ from the other CEHs at some point within the 13.5 kb stretch just centromeric to *HLA-DQB2* between the SNPs DC9-6 and DC10-2. The vast majority of all other common DR4,DQ8 haplotypes (33 of 34; 97%) remained identical to SSTO at amplicon DC10. (Three common haplotypes were not sequenced at DC9 or DC10 but retained identity to the dominant sequence at the next amplicon (DC13) and through at least *TAP2*.)

However, 11.2 kb centromeric to DC10 (at DC13), the SSTO sequence differed from all sequenced DR4,DQ8 haplotypes at two or more of six SNPs. (One of the previously identical DR4,DQ8 haplotypes was not sequenced at DC13 but remained identical to the others centromeric to DC13.) Thus, the centromeric breakpoint where SSTO no longer represented any DR4,DQ8 haplotype was within an 11.2 kb region between amplicons DC10 and DC13 (Table S4). The breakpoint of SSTO representation of its group was the most telomeric breakpoint of the eight MHP sequences.

**Class II fixity of DR4,DQ8 CEHs.** We resequenced six different DR4,DQ8 CEHs (Table S4) in multiple examples for most but only one example for one CEH. Below, we report our results for each CEH individually. They are shown in the order in which they differed (i.e., broke away centromerically) from the majority of DR4,DQ8 haplotypes.

**B49,DR4,DQ8.** We sequenced a single haplotype with the classical MHC markers of the CEH [*C*07:01, B*49:01, SC01, DRB1*04:05, DQB1*03:02*]. Table S4 provides the SNP/DIP sequence of this haplotype, which differed from SSTO and all other DR4,DQ8 CEHs at and centromeric to *HLA-DQA2*. Although we could not determine to what extent (if any) this sequence was representative of this CEH from the data presented here, from *HLA-DQA2* through *TAP2* this sequence was identical to the dominant B44,DR7 CEH sequence at every position sequenced (Table S4). Beginning near *HLA-DMB*, however, this haplotype had a unique sequence.

**B44,DR4,DQ8.** We sequenced six haplotypes with the MHC markers of this CEH. All six contained the *HLA-DRB1*04:01* and *HLA-DQB1*03:02* alleles. Two of the haplotypes contained the SC30 complotype and four contained SC31. This *C4B* microvariation did not correlate with sequence differences among the six haplotypes at or centromeric to *HLA-DQA2*. From *HLA-DQA2* through 16.4 kb centromeric to *HLA-DQB2* (amplicon DC10), three of the six haplotypes (50%) were identical to SSTO and the vast majority of all DR4,DQ8 haplotypes. These three haplotypes remained identical to the dominant DR4,DQ8 sequence (but different from SSTO) through *HLA-DOB* (DOB4 amplicon; Table S5). Just 2 kb centromeric to *HLA-DOB* (at DOB5), one sequence became different. Within intron 9 of *TAP2* (at amplicon DOB9), the remaining two haplotypes lost sequence identity to one another, but one retained the dominant DR4,DQ8 sequence.

The other three B44,DR4,DQ8 haplotypes (50%) had a different sequence. Their common sequence from *HLA-DQA2* through the DC10 amplicon was identical to that of the DBB cell line. At DC13 and centromeric through *ZBTB22*, two of these three haplotypes had a unique common sequence differing from that of DBB, while the third haplotype continued to bear the same DBB sequence through *HLA-DOB* (DOB4 amplicon) and then became different (Table S5). Therefore, two of the original six B44,DR4,DQ8 haplotypes (33%), both of which were typed as *HLA-DPB1*04:01*, maintained a fixed and identical sequence through *ZBTB22*, approximately 650 kb centromeric to *HLA-DQB1*. Table S4 presents the results throughout the region for this alternative dominant B44,DR4,DQ8 CEH sequence.

**B62,SC33,DR4,DQ8.** We sequenced seven haplotypes with markers of the CEH [*HLA-C*03:04, B*15:01, SC33, DRB1*04:01, DQB1*03:02*]. Six of these haplotypes were typed at high resolution and had *HLA-DRB1*04:01* and *HLA-DQB1*03:02*. All seven contained the complotype SC33. An eighth haplotype contained all of the markers of this CEH except its complotype was SC31. From *HLA-DQA2* through approximately 2.6 kb centromeric to *HLA-DQB2* (DC9 amplicon), all eight haplotypes remained identical to SSTO, but approximately 16.4 kb centromeric to *HLA-DQB2* (at DC10), seven of the eight (88%) haplotypes remained identical to each other but differed from the SSTO sequence and the vast majority of all DR4,DQ8 haplotypes at eight of 21 SNPs. The remaining haplotype retained the same sequence as SSTO.

The seven haplotypes with the dominant B62,SC33,DR4,DQ8 sequence within amplicon DC10 remained identical to one another through 2.1 kb telomeric to *HLA-DMB* (amplicon DMP1; Table S5). The fixity of this CEH decreased from 88% at DMP1 to 75% through exon 2 of *HLA-DMA* (DMP4 amplicon) and further decreased to 63% between intron 1 of *HLA-DMA* (DMP5 amplicon) and 1 kb telomeric to *HLA-DOA* (DMP12 amplicon). Four of the eight (50%) haplotypes remained identical to one another within *HLA-DOA* (DMP13 amplicon) and three (38%) retained the dominant sequence of this CEH through *DAXX* (DMP18 amplicon). Table S4 presents the results throughout the region for this last dominant sequence.

**B38,SC21,DR4,DQ8.** We sequenced nine haplotypes with markers of the CEH [*HLA-C*12:03, B*38:01, SC21, DRB1*04:02, DQB1*03:02*]. Two of the haplotypes were not complotyped nor typed at *HLA-DQB1* but were otherwise identical. Four haplotypes were typed at 4-digit resolution at *HLA-DRB1*, and all contained the *HLA-DRB1*04:02* allele typical of this CEH. All six of the haplotypes typed at *HLA-DQB1* at 4-digit resolution had *HLA-DQB1*03:02*. At *HLA-DPB1*, all six haplotypes typed at 4-digit or higher resolution contained *HLA-DPB1*04:01* or *HLA-DPB1*04:01:01*. From *HLA-DQA2* through amplicon DC10, all nine haplotypes remained identical to the SSTO sequence. At amplicon DC13, all B38,SC21,DR4,DQ8 haplotypes remained identical to each other and the majority of other DR4,DQ8 haplotypes which had been identical to the SSTO sequence up to the DC10 amplicon, but were now different from SSTO.

The B38,SC21,DR4,DQ8 CEH sequence remained identical to most other DR4,DQ8 haplotypes through *HLA-DOB* and much of *TAP2*, but this CEH sequence began to differ from other DR4,DQ8 sequences within intron 3 of *TAP2* (amplicon DOB12; Table S4, Table S5). At and centromeric to that point, all nine B38,SC21,DR4,DQ8 haplotypes remained identical in sequence through 18.6 kb centromeric to *RING1* (amplicon CTB12). At 9 kb telomeric to *VPS52* (amplicon CTB13), one of the nine haplotypes had a different sequence, but the remaining eight (88%) haplotypes remained identical to one another through *DAXX*. Table S4 presents the dominant sequence throughout the region. The B38,SC21,DR4,DQ8 CEH had the highest fixity frequency at *DAXX* of all the CEHs we studied.

**B60,SC31,DR4,DQ8.** We sequenced 10 haplotypes with the classical markers of the CEH [*HLA-C*03:04, B*40:01, SC31, DRB1*04:04, DQB1*03:02*]. Six of the 10 haplotypes were typed at 4-digit resolution at *HLA-DRB1* and *HLA-DQB1*, and they all contained the *HLA-DRB1*04:04* allele typical of this CEH as well as *HLA-DQB1*03:02*. All but one of the haplotypes was typed at serological or low resolution at *HLA-B* as *HLA-B*40.* *HLA-B*40* alleles split largely into two *HLA-B* specificities: HLA-B60 and HLA-B61. When typed for this split, these haplotypes all typed as HLA-B60. The remaining haplotype not typed at *HLA-B* contained the *DRB1*04:04* allele. Only two of six (33%) haplotypes typed at *HLA-DPB1* shared an allele (*HLA-DPB1*03:01*).

From *HLA-DQA2* through *TAP2* (amplicon DOB12), nine of the 10 (90%) B60,SC31,DR4,DQ8 haplotypes were identical to most DR4,DQ8 haplotypes (excepting for break points in the DR4,DQ8 CEHs described above). However, this CEH sequence differed from other DR4,DQ8 haplotype sequences 2.1 kb telomeric to *HLA-DMB* in seven of the nine (78%) previously identical haplotypes (Table S4, Table S5). Therefore, at least seven of 10 (70%) B60,SC31,DR4,DQ8 haplotypes had a unique consensus sequence which began at some point within the 100 kb region between *TAP2* and 2.1 kb telomeric to *HLA-DMB*. Centromerically, all seven haplotypes had the same sequence through 2.6 kb telomeric to *BRD2* (amplicon DMP8). (Only five of the seven haplotypes were sequenced at amplicon DMP7, and all five had the same sequence in that region. At amplicon DMP9, only three of the seven haplotypes were sequenced and they showed an identical sequence.) At amplicon DMP10, only four of the seven haplotypes were sequenced and they showed identical sequences except for a microvariation at the *BRD2* second DIP (ID #204; Table S2). We concluded that seven of the 10 (70%) B60,SC31,DR4,DQ8 haplotype sequences remained essentially identical to one another through the 3’ UTR of *BRD2* (amplicon DMP10; Table S5).

We only performed incomplete sequencing of the B60,SC31,DR4,DQ8 haplotypes for the amplicons DMP11 through DMP17, but the results suggested a steady decline in fixity among this group of haplotypes from 70% at *BRD2* to 50% at *HLA-DOA* to 30% at *HLA-DPA1*. *HLA-DPB1* typing of these haplotypes suggested that fixity decreased to 20% at that locus. Sequence identity was maintained at 20% centromeric to *DAXX*. Table S4 presents the sequence throughout the region for this dominant sequence.

**B62,SB42,DR4,DQ8.** We sequenced five haplotypes with all of the markers of the CEH [*HLA-C*03:03, B*15:01, SB42, DRB1*04:01, DQB1*03:02*]. One of the five haplotypes was not typed at *C2*, but contained the other complotype alleles of this CEH. We also sequenced two additional haplotypes containing the centromeric (complotype and DR/DQ alleles) of this CEH. Six of these seven haplotypes were typed at 4-digit resolution at *HLA-DRB1* and *HLA-DQB1*, and all contained *HLA-DRB1*04:01* and *HLA-DQB1*03:02* alleles. From *HLA-DQA2* through *TAP2* (amplicon DOB12) all seven (100%) B62,SB42,DR4,DQ8 haplotypes remained identical to each other and most of the remaining DR4,DQ8 haplotypes (excepting for the break points in the DR4,DQ8 CEHs described above).

All seven (100%) B62,SB42,DR4,DQ8 CEH sequences differed from most other DR4,DQ8 haplotype sequences (except the B38,SC21,DR4,DQ8 haplotypes) at the amplicon 2.1 kb telomeric to *HLA-DMB* (DMP1; Table S4). Therefore, in the approximately 100 kb region containing both amplicons DOB12 and DMP1, the B62,SB42,DR4,DQ8 haplotypes had a unique consensus sequence. Centromerically, all seven (100%) B62,SB42,DR4,DQ8 sequences remained identical through *BRD2* (at amplicon DMP10; Table S5).

Throughout the region extending from *HLA-DOA* to *HLA-DPB1*, the fixity of this CEH declined steadily. Only five of the B62,SB42,DR4,DQ8 haplotypes were sequenced at amplicons DMP11, DMP12 DMP13 and DMP14, and all seven were sequenced at DMP15 and DMP16. Given the incomplete sequencing at amplicons DMP11 through DMP14, the CEH fixity in that region could only be estimated to range between 29% and 57%. By *HLA-DPA1* (amplicon DMP14), only two of the seven (29%) haplotypes retained a common sequence. This sequence identity continued through amplicon CTB18 at *DAXX*. That dominant sequence for the B62,SB42,DR4,DQ8 CEH is shown in Table S4. One of those two fixed haplotypes was typed as *HLA-DPB1*04:01*.

**Sequence fixity of DR13,DQ6 haplotypes**

APD is homozygous for the haplotype (*HLA-C*06:02, B*40:01, DRB1*13:01, DQB1*06:03*). This haplotype does not exist among our 2675 normal Boston haplotypes [21]. We sequenced 13 (*HLA-DRB1*13, DQB1*06*) ("DR13,DQ6") haplotypes. Eight of these were (*HLA-DRB1*13:02, DQB1*06:04*), of which five also contained at least the complotype markers and of which at least four also contained both at least one class I marker and the complotype markers of the CEH [*HLA-C*03:04, B*40:01, SC02, DRB1*13:02, DQB1*06:04*] (“B60,DR13”), which is the only B60,DR13 CEH in our database [21]. (*HLA-B*40:01* alleles belong to the HLA-B60 specificity.) The other five haplotypes we sequenced were one each of (*HLA-DRB1*13:01, DQB1*06:04*) and (*HLA-DRB1*13:02, DQB1*06:05*), both of which contained the FC31 complotype, two (*HLA-DRB1*13:01, DQB1*06:03*) haplotypes (one with SC30 and one with SC31) and one haplotype that was only typed at low (2-digit) resolution. Sequencing of these 13 DR13,DQ6 haplotypes yielded six different *HLA-DQA2* haplotypes. The most frequent *HLA-DQA2* sequence among the 13 was found in five haplotypes, four of which were members of the B60,DR13 CEH mentioned above. APD did not represent that CEH in the regions we resequenced, and we shall describe that CEH sequence in a later report.

The *HLA-DQA2* sequence for APD was found in only one of the 13 DR13,DQ6 haplotypes we sequenced. That haplotype was (*HLA-B*15, FC31, DRB1*13:01, DQB1*06:04*). We report its sequence and that of APD in Table S4. The two sequences remained identical to one another through at least intron 3 of *TAP2* (amplicon DOB12). The previously identical sequence differed from APD at every SNP 2.1 kb telomeric to *HLA-DMB* (DMP1; Table S4) but was otherwise identical at amplicons DMP2-6. Within and near *HLA-DMB* and *HLA-DMA* (amplicons DMP1-6), no other haplotype we sequenced had the APD sequence, and only two other haplotypes (both non-standard haplotypes from other groups) had the same sequence as the DR13,DQ6 haplotype we report in Table S4. The APD sequence in amplicons DMP7 through DMP13 has not been reported, but its sequence near and in the HLA-DP genes differed from the other DR13,DQ6 haplotype we report (Table S4).
